# Genome wide *in-silico miRNA* and target network prediction from stress responsive Horsegram (*Macrotyloma uniflorum*) accessions

**DOI:** 10.1038/s41598-020-73140-x

**Published:** 2020-10-14

**Authors:** Jeshima Khan Yasin, Bharat Kumar Mishra, M. Arumugam Pillai, Nidhi Verma, Shabir H. Wani, Hosam O. Elansary, Diaa O. El-Ansary, P. S. Pandey, Viswanathan Chinnusamy

**Affiliations:** 1 Division of Genomic Resources, ICAR-National Bureau Plant Genetic Resources, PUSA Campus, New Delhi, 110012 India; 2 Department of Plant Breeding and Genetics, Agricultural College and Research Institute, Tamil Nadu Agricultural University, Killikulam, Vallanadu, Tamil Nadu 628252 India; 3 Principal Scientist (Education Planning and Home Science), Agricultural Education Division Krishi Anusandhan Bhawan I, Indian Council of Agricultural Research, PUSA Campus, New Delhi, 110 012 India; 4 Mountain Research Centre For Field Crops, Khudwani Anantnag-192101, Sher-E-KashmiR University of Agricultural Sciences and Technology of Kashmir, Badgam, J&K India; 5 Plant Production Department, College of Food and Agricultural Sciences, King Saud University, P.O. Box 2455, Riyadh, 11451 Saudi Arabia; 6 Floriculture, Ornamental Horticulture, and Garden Design Department, Faculty of Agriculture (El-Shatby), Alexandria University, Alexandria, 21545 Egypt; 7 Precision Agriculture Laboratory, Department of Pomology, Faculty of Agriculture (El-Shatby), Alexandria University, Alexandria, Egypt; 8 Indian Council of Agricultural Research (ICAR), PUSA, New Delhi, 110 012 India; 9 Division of Plant Physiology, Indian Agricultural Research Institute, New Delhi, 110012 India; 10 Present Address: Department of Biology, University of Alabama at Birmingham, Birmingham, AL 35294-1170 USA

**Keywords:** Agricultural genetics, Genetic markers, Plant genetics, Biotechnology, Computational biology and bioinformatics, Genetics, Molecular biology

## Abstract

Horsegram (*Macrotyloma uniflorum* (Lam.) Verdc.) is a drought hardy food and fodder legume of Indo-African continents with diverse germplasm sources demonstrating alternating mechanisms depicting contrasting adaptations to different climatic zones. Tissue specific expression of genes contributes substantially to location specific adaptations. Regulatory networks of such adaptive genes are elucidated for downstream translational research. *MicroRNA*s are small endogenous regulatory RNAs which alters the gene expression profiles at a particular time and type of tissue. Identification of such small regulatory RNAs in low moisture stress hardy crops can help in cross species transfer and validation confirming stress tolerance ability. This study outlined prediction of conserved *miRNA*s from transcriptome shotgun assembled sequences and EST sequences of horsegram. We could validate eight out of 15 of the identified *miRNAs* to demonstrate their role in deficit moisture stress tolerance mechanism of horsegram variety Paiyur1 with their target networks. The putative mu*miRs* were related to other food legumes indicating the presence of gene regulatory networks. Differential *miRNA* expression among drought specific tissues indicted the probable energy conservation mechanism. Targets were identified for functional characterization and regulatory network was constructed to find out the probable pathways of post-transcriptional regulation. The functional network revealed mechanism of biotic and abiotic stress tolerance, energy conservation and photoperiod responsiveness.

## Introduction

*MicroRNAs* (*miRNAs*/ *miRs*) are 19–24 nucleotides long endogenous molecular bigwigs in post-transcriptional gene regulatory networks^1^. These *miR* genes are capped, polyadenylated and spliced like other RNA polymerase II transcripts. The mature *miRs* are located in a hairpin structure within the primary transcript (pri-transcript) and are preprocessed by at least two RNase mediated steps to mature *miRNA*. *Pri-miRNAs* range from 50–100 nucleotides that coil into hairpin loop structures encompassing paired stems and unpaired loops^2^. Intensive investigation over decades has enriched the *miRNA*’s knowhow in biogenesis and explicit regulatory machinery. Plant *miRNA* precursors are less conserved, whereas mature *miRNA*s are conserved at higher magnitude in comparison to animal *miRNA*s^3^. Hence, exploring conserved *miRNA*s makes sense to identify the potential *miRNA*s from flora with targeted traits.

*Macrotyloma uniflorum* (Lam.) Verdc. (Horsegram) originated from South-Western India is a multipurpose pulse crop mainly cultivated for providing nutritional security in the form of food, livestock supplement and green manure in India and Africa^4–6^. Being a diploid (2n = 20, 22 or 24], short duration (120–180 days to maturity) plant species and adapted to grow on wide range of agro-climatic conditions, horsegram can be weighed as appropriate model for moisture stress tolerance genes/QTLs investigation challenging undernourishment in drought prone regions^6,7^. Further, it can be envisaged as nutraceuticals, forage crop^8^ and anti-calcifying inhibitors^9^. Recently, substantial improvement in accumulation of EST^5,10^ and transcriptome data^11^ has compelled its position as a future crop with versatile utility. The present study details the comprehensive computational approach^12–14^ to predict *miRNA*s fromavailable ESTs and Transcriptome Shotgun Assembly (TSA) sequences of horsegram based on *miRNA* homolog search. Potential pathways contributing to drought tolerance is studied as inherent trait of horsegram, which could have common or divergent gene regulatory networks^6,10,15^.

Flora without whole genome sequences are having alternate resource sequence sources in public databases such as Genome Survey Sequences (GSS), Expressed Sequence Tags (EST) and Bacterial Artificial Chromosome (BAC) sequences rendering plentiful resources to mine conserved microRNAs^16–18^. The database *miR*Base hosts 8746 reported *miRNA*s belonging to four phyla of plant species enlisted both mature and precursor *miRNA*s (https://www.miRbase.org/) (Release 21, June 2014). Plant *miR*s share functional similarity with small interfering RNAs (siRNAs) in guided target cleavage as microRNA targets sites of coding *mRNA* sequences^3,19^. Currently, the data generated from next generation sequencing (NGS) studies have been employed in *miRNA*s prediction and their impact on multiple traits in related species. The *in-silico* homology based prediction of *miRNA* is advantageous and orthologs of previously reported *miRNA*s could be deciphered with their evolutionary significance among species^20–22^. In recent years, the multi-faceted functionalities of *miR*s in plants are being understood effectively despite the fact that *miRNA*s are less studied in plants.

This is the first report in horsegram with comprehensive analysis of conservation and phylogeny of *miR*s. Previously, a comprehensive study was performed in horsegram from ESTs in predicting eight novel *miR*s^11^. Here we report differential expression of identified *miR*s through a stringent *in-silico* schema elucidating conserved *miRNA*s, their characterization, validation with their target annotation and networking.

## Materials and methods

### Query and reference datasets

Totalling 27,997 shotgun assembled contigs of transcriptome were collected from TSA sequence set: GANR01000001-GANR01027997 from European Nucleotide Archive (www.ebi.ac.uk/ena), SSH-Mu library of moisture stressed cDNA of Macrotyloma (https://www.ncbi.nlm.nih.gov/nucest NCBI dbEST ID 75866463 from) and 1008 ESTs were downloaded (www.ncbi.nlm.nih.gov/dbEST/) from publically available NCBI EST database to represent query sequences for *miRNA* homolog search. About 8496 *miR*Base mature Viridiplantae *miRNA*s were used (https://www.miRbase.org) (Released 21: June, 2014) database^23^ in the present investigation and clustered by CD- HIT-EST, with threshold value of 100^24^. Out of 3777 clustered sequences, only non-redundant *miRNA*s were used as reference *miRNA*s for finding the homologs in *M. uniflorum* (Lam.) Verdc. candidate sequences to create a local nucleotide sequence database. To elucidate likely role of *miRNA*s involved in drought stress tolerance trait, the predicted microRNAs were screened for target genes listed in DroughtdB database (https://pgsb.helmholtz-muenchen.de/droughtdb/).

### Bioinformatics tools employed

For conserved *miRNA* prediction of horsegram candidate sequences from Viridiplantae *miRNA*s reported in *miR*Base database, NCBI BLAST version 2.2.27^25^ an alignment tool was used (https://ftp.ncbi.nih.gov/blast/executables/blast+). The representative sequences of all plant *miRNA*s were obtained after clustering with CD-HIT-EST with threshold value of 100^24^. MFOLD online tool^26^ was (https://unafold.rna.albany.edu/?q=mfold) used for pre-*miRNA*s secondary structures prediction. The *miRNA* targets were deduced using psRNAtarget server^27^. Online circoletto^28^ tool was used to illustrate the *miRNA*s and its target genes by circos plot^29^. The phylogenetic analysis of predicted *miRNA*s with their closely related *miRNA* families was performed with MEGA7^30^.

### Computational prediction of potential *miRNA* homologs

The prediction of *miRNA* homologs was performed using TSA and EST sequences of *M. uniflorum* available at NCBI and EBI databases. The plant *miRNA*s were obtained from NCBI was clustered and the representative sequences generated were employed as reference *miRNA* database using BLAST-2.2.27+. The query sequence consisting of TSA and EST of horsegram were subjected to nucleotide blast (blastn) against reference sequences of plant *miRNA*s with the following set parameters: (i) word match size-7; (ii) length of mature *miRNA* sequence ≥ 18 nt without gap; (iii) mismatch range- 0–2 (iv) e-value-0.1. The filtered sequences were utilized to find out the coding and non-coding candidate sequences by performing blastx against non-redundant protein database. The protein coding regions were removed whereas the non-coding regions were exploited for secondary structure prediction and validation.

### Secondary structure prediction and validation

With maximum stringency only non-coding sequences were subjected to Zuker’s folding algorithm based secondary structure prediction in MFold^26^. A sliding window of 100nt from ~ 80nt upstream and ~ 80nt nucleotide downstream of the mature *miRNA* were set to find the precursors^33^ with parameters set as: (a) Folding temperature- 37 °C, (b) Ionic conditions of 1 M NaCl without divalent ions, (c) linear RNA sequence, (d) percent sub-optimality number of 5 and (e) maximum interior/bulge loop size-30. As reported elsewhere, to validate the structures of pre-*miR*s adjusted minimal folding free energy (AMFE) and minimal folding free energy index (MFEI) were calculated^34^ (Table 1).

**Table 1:** Characteristics of predicted precursor *miRNA*s of horsegram.

TSA ID	MFP	LT	LT	LP	AU%	GC%	A	C	G	U/T	MFE	AMFE	FEI
GANR01006328	*miR*482	355	355	119	57.99	42.01	38	28	22	31	− 42.01	− 31.34	0.74
GANR01007318	*miR*482	460	460	130	39.24	40.76	38	21	32	39	− 40.76	− 35.61	0.87
GANR01008465	*miR*156	609	609	100	50	50	31	19	19	31	−50	−39	0.78
GANR01009673	*miR*2673	775	775	118	53.39	46.61	28	17	38	35	− 46.61	− 26.77	0.57
GANR01016903	*miR*5653	2143	2143	119	52.11	47.89	36	25	32	26	− 47.89	− 28.49	0.59
GANR01019897	*miR*156	670	670	107	56.08	43.92	30	26	21	30	− 43.92	− 26.82	0.61
GANR01022354	*miR*1507	950	950	91	54.45	45.05	31	23	18	19	− 45.05	− 36.04	0.8
GANR01022920	*miR*168	515	515	122	41.81	58.19	19	36	35	32	− 58.19	− 48.85	0.83
GANR01023649	*miR*2118, *miR*482	644	644	106	60.38	39.62	36	19	23	28	− 39.62	29.05	0.7
GANR01024080	*miR*390	576	576	102	56.87	43.13	24	24	20	29	− 43.13	44.41	1.03

MFP, microRNA family/families present in stem-loop structure; LT, length of TSA; LP, length of precursor *miRNA*; MFE -minimal folding free energy (−Kcal/mol); AMFE, adjusted minimal folding (−Kcal/mol); MFEI, minimal folding free energy index.

The secondary structures were screened for^35^: (i) Less than three nucleotide substitutions within predicted mature *miRNA* to reference *miR*, (ii) The candidate sequence must fold into an appropriate and proper stem-loop hairpin secondary structure, (iii) The localization of mature *miRNA* must be in one arm of the stem-loop structure, (iv) Less than six mismatches between mature *miRNA* and its corresponding star sequence (miRNA*) and (v) The secondary structure of putative *pre-miR* must contain high negative MFE and MFEI levels. Putative horsegram *miRNA*s were designated based on the standard nomenclature system^23,36^.

### Phylogeny of horsegram *miRNA*

To enumerate the conserved nature and its phylogenetic relationship of *miR*s and its precursors were aligned with reported plant *miRNA*s using BLASTn with e-value, maximum mismatch and hits number set as 10, 3 and 5 respectively. The homologous precursor *miRNA*s were aligned to predicted *miRNA*s with Phylogeny.fr web tool (https://www.phylogeny.fr/index.cgi)^37^ by integrating multiple sequence alignment tools, MUSCLE^38^ and GBlocks^39^ to refine the alignment; phylogenetic tree construction by PhyML^40^ and Tree rendering by TreeDyn^41^. The alignment output is used in MEGA7^30^ for interpretation of molecular clock to estimate interfamily evolution.

### Phylogenetic analysis

The analysis involved 146 nucleotide sequences. All positions containing gaps and missing data were eliminated. Evolutionary analyses were also conducted in MEGA7^30^.

### Functional annotation of *miRNA* targets

As annotations are not available for horsegram, the closely related soybean was used as a reference to annotate *miR* targets of the predicted *miRNA*s. Putative identified query *miRs* were hit against horsegram *mRNA* sequences using psRNATarget tool^27^. The parameters set target prediction were (i) 9–11 nucleotide mismatch range for translational inhibition, (ii) maximum 4 mismatches without gap at complementary site, (iii) multiplicity of target sites -2, (iv) maximum expectation value -3 and (v) number of hits -10. The homologs of putative *miRNA*s and their targets were represented in *Glycine max* genome as circos plot to demonstrate multiple targets of identified putative *miRNAs* in horsegram^28^. In addition, to elucidate the role of identified *miR*s in drought stress response, specific *miRNA* target genes among drought tolerance contributing genes reported in Droughtdb (https://pgsb.helmholtz-muenchen.de/droughtdb/) were confirmed. The genes reported for horsegram are insufficient, thus, *A. thaliana* was used as reference organism to determine gene targets for the new *miRNA*s predicted. The identified putative *miRNA*s were used as query against the *A. thaliana* DFCI gene index (AGI) release 15 and *A. thaliana* TAIR10, cDNA, removed *miRNA* gene (release date 14th December 2010) using psRNATarget tool. The parameters set for the target gene prediction were as follows: (i) range of central mismatch for translational inhibition 9–11 nucleotide; (ii) a maximum of 64 mismatches without gaps at complementary site; (iii) multiplicity of target sites 2; (iv) maximum expectation value 3 and (v) Number of hits -5. Similarly, to understand the specific possibilities of putative horsegram *miRNA*s involved in drought stress, the drought genes were downloaded from Droughtdb in order to search for drought *miRNA* target genes. The visual representation of *miRNA* and its targets was represented by drawing circus plot to show the multiple targets of horsegram microRNAs predicted.

### Functional annotation and metabolic pathway analysis

**Figure 1:**
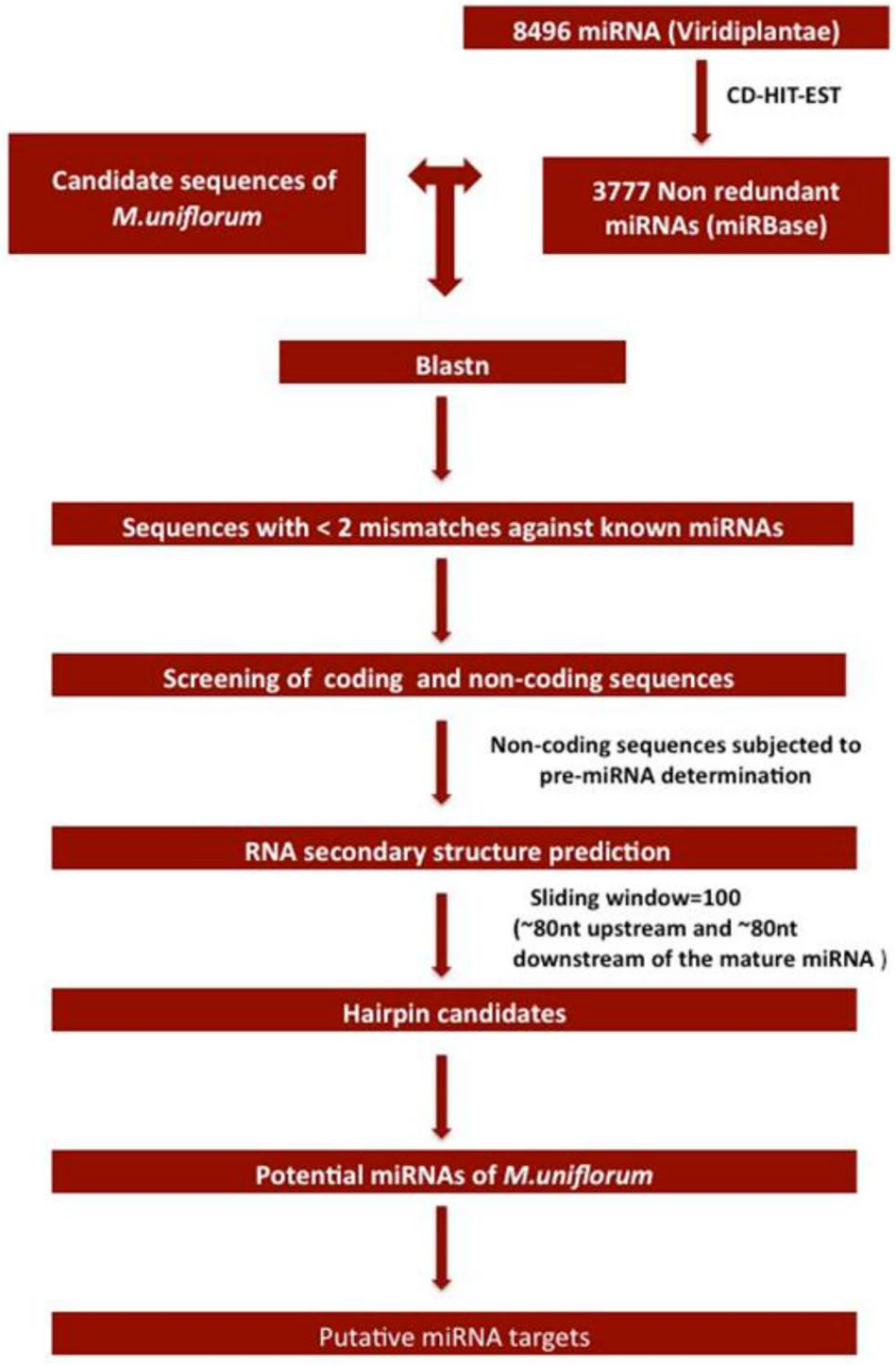
Pipeline for prediction of *miRNA*s and its *in-silico* validation. From non-coding seqeuences and secondary structure analyses miRs were predicted in-silico as depicted in this pipeline.

Mercator^42^ AgriGO^43^ and B2g^44^ were used to determine the functional roles and to classify Gene Ontology (GO) terms into molecular functions, biological processes and cellular components. Further, corresponding pathways were mined through KASS server, which assigns KEGG Orthology (KO) terms and employs KEGG pathways for *miRNA* target genes. The simplified schema of workflow adopted in present investigation for *miRNA* prediction is given in Fig. 1.

### Quantitative expression analysis of identified miRNAs

Stress responsive horsegram germplasm were identified from the germplasm core developed as described earlier (6). Selected stress responsive variety Paiyur 1 was raised in glass house under control and moisture stress conditions. Plants were maintained at field condition for control and at temporary wilting point for stress condition. Leaf tissues of control and stress plants were collected and fixed in RNALater and stored at − 80 °C till RNA isolation.

High quality RNA samples were extracted from 100 mg (wet weight) leaf tissue using Plant RNA Easy mini spin coloumn kit following the manufacturer’s guidelines (Qiagen). Quantity and quality of the RNA was quantified with the advanced Qiaexpert.

RNA samples of one microgram each were 3′ polyadenylated using Poly A RNA polymerase (Sigma Aldrich). Forward primers sequences of all 15 miRs and a common Poly T reverse primers were designed (Supplementary material 1).

Quantitative rtPCR using One Step PrimeScript™ RT-PCR Kit with SYBR green (Takara) was performed for all 15 identified miRs. qPCR capturing was conducted using the Illumina Eco RT PCR machine.

For qPCR cycling conditions set were 50 °C for 10 min for cDNA sysnthesis; 95 °C for 3 min for polymerase activation followed by 45° cycles of 95 °C 15 s, 60 °C for 30 s capturing with SYBR green filter at end of each cycle.

## Results

### *miRNA*s in *M.uniflorum*

**Figure 2:**
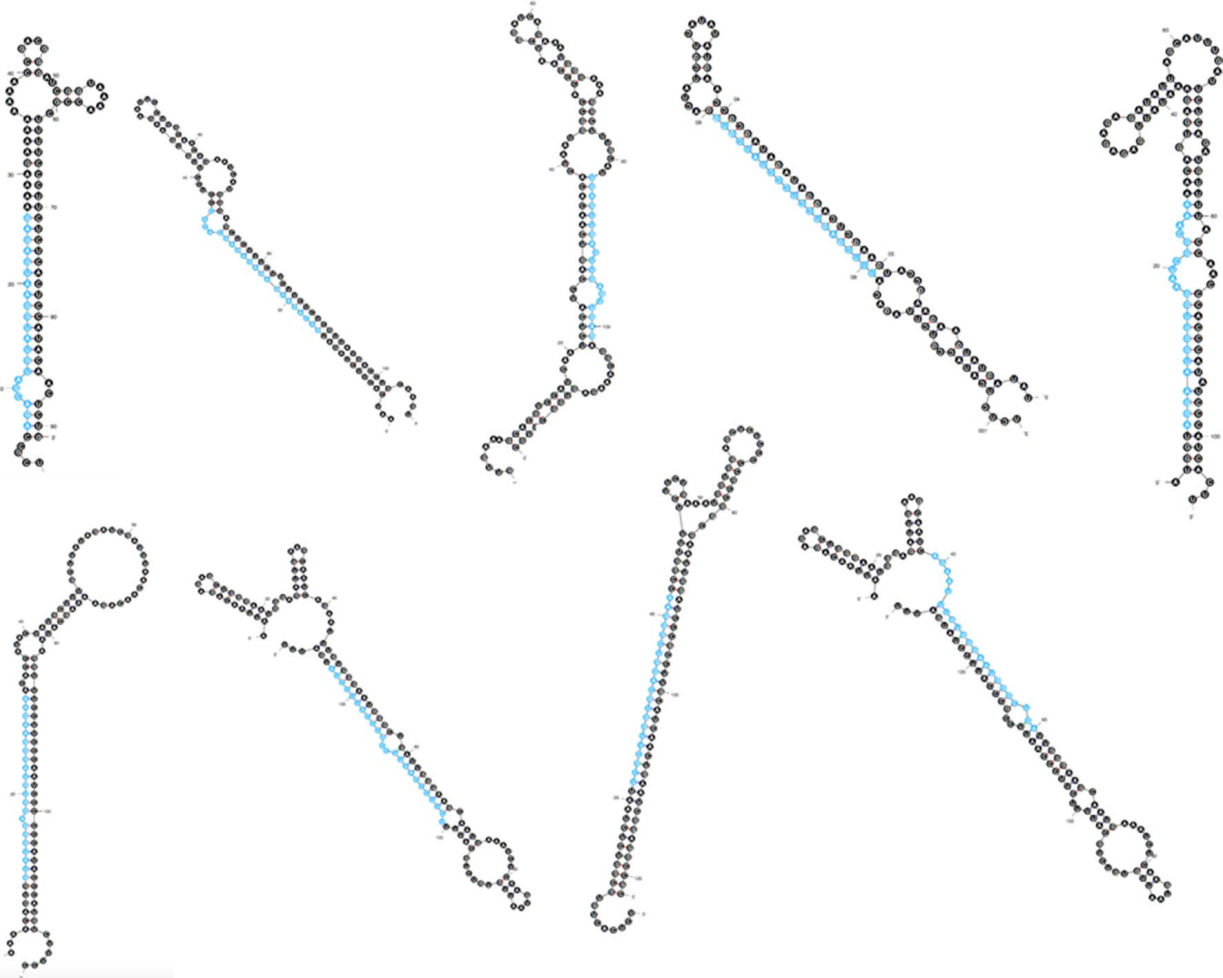
Secondary structures of predicted *miRNA* precursors of horsegram using MFOLD tool^26^.

**Figure 3:**
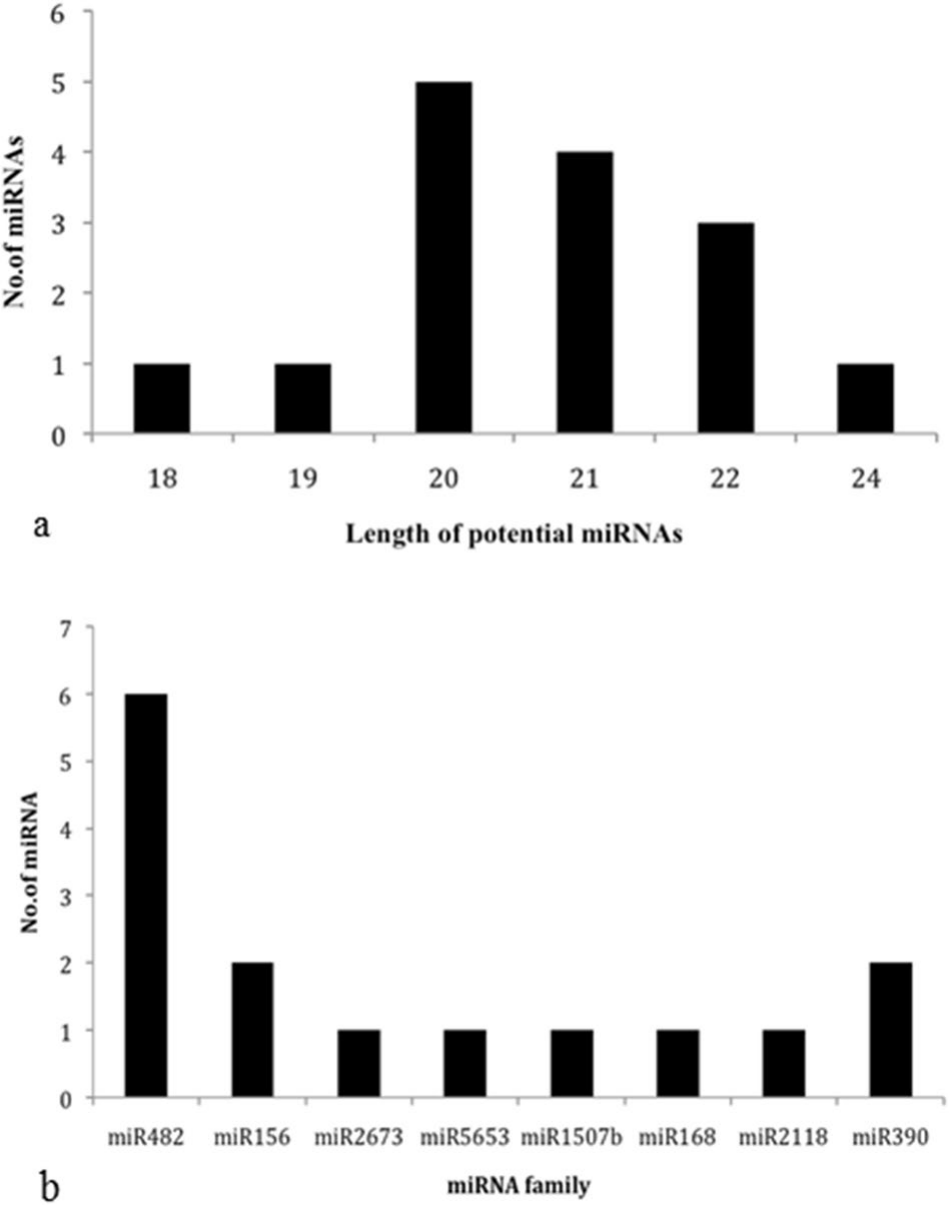
Identified *miRNA*s with (**a**) length variation and (**b**) family size in horsegram. The differentially expressed *miR*s were found to have more members in horsegram indicating network of *ncRNA*s playing key role in regulating stress tolerance mechanism.

Significant *miRNA* homologs within reported 8496 *miRNA*s were identified by executing nucleotide blast (BLASTn) with 27,997 TSA contigs, SSH-Mu library sequences of moisture stressed horsegram cDNA (NCBI dbEST ID 75866463) and 1008 EST sequences as query resulted in 16 ESTs and 6303 TSA hits (E value 0.1) for further analysis. Stringent filtering (mismatch < 3 and length > 17) was incorporated to narrow down the hits to 5807 (8 ESTs and 5799 TSA) sequences for putative candidate sequences identification with non-coding regions. Based on coding potential, 214 noncoding sequences (7 ESTs and 207 TSA) were filtered out to predict ten distinct pre-*miR*s coding for 15 conserved mature *miR*s (Tables 1 and 2) clustering into nine different *miR*-families (Figs. 2 and 3).

**Table 2:** Details of the predicted horsegram *miRNA*s (tentative names given to putative predicted *miRNA*s. Submitted to *MiR*Base).

Horsegram *miRNA*	*miRNA* homolog	Mature *miRNA* sequence	LM	NM	Loc	Strand
mun-*miR*482d-3p	gma-*miR*482d-3p	gguaugggagguguagggaaga	22	0	3′	−
mun-*miR*482b-5p	gma-*miR*482b-5p	uuccuucccaauccccccaua	21	0	5′	−
mun-*miR*482a-5p	gma-*miR*482a-5p	agaauuugugggaaugggcuga	22	0	5′	+
mun-*miR*482-5p	pvu-*miR*482-5p	ggaaugggcugauugggaagca	22	0	5′	+
mun-*miR*482a-3p	gma-*miR*482a-3p	ucuucccaauuccgcccauuccua	24	0	3′	+
mun-*miR*156r	gma-*miR*156r	ugcucucuaucuucugucag	20	0	5′	−
mun-*miR*2673a	mtr-*miR*2673a	ucuuccucuuccucuucc	18	0	3′	+
mun-*miR*5653	ath-*miR*5653	ugaguugaguugaguugag	19	1	3′	+
mun-*miR*156k	osa-*miR*156k	gacagaagagagagagcaca	20	0	5′	+
mun-*miR*1507a	gma-*miR*1507a	agacgauguauggaaugaga	20	0	3′	−
mun-*miR*168a-5p	ath-*miR*168a-5p	ucgcuuggugcaggucgggaa	21	0	5′	+
mun-*miR*2118	pvu-*miR*2118	aggaauggguggaaucggcaa	21	0	3′	−
mun-*miR*482a	sly-*miR*482a	aggaauggguggaauuggaaa	21	2	3′	−
mun-*miR*390b-5p	gma-*miR*390b-5p	gugcuaucccuccugagcuu	20	0	5′	−
mun-*miR*390a-5p	ath-*miR*390a-5p	gcgcuaucccuccugagcuu	20	1	5′	−

LM, Length of the mature *miRNA.*

### Secondary structure prediction and validation

The predicted *miRNA*s were analyzed for various structural features to distinguish from other small RNAs such as *tRNA*s, *rRNA*s and *mRNA*s (Table 1 and Fig. 2). Most crucial characteristic feature of stable secondary structure is its minimal folding energy (MFE) ranging from 37.3 to 59.6 (−kcal/mol) for ten *in-silico* predicted pre*miRNA*s. The MFE of the precursor *miRNA* was retained low to achieve thermodynamic stability^45^. Owing to their sequence length polymorphism, *pre-miRNAs* characterization was based only on MFEI (Minimum free energy index) following previous reports^22,46^. The MFEI ranges from 0.57 to 1.03 for ten *in-silico* predicted pre-*miRNA*s (Fig. 2). The (A + U) % of precursor horsegram *miRNA*s ranges from 39.24–60.38 satisfies the criteria included by Zhang et al.^18^. Nucleotide distribution (A = 31.6%, U = 32.31%, G = 26% and C = 23.8%) of the predicted pre-*miR* are heterogeneous as given Tables 1 and 3. On an average pre-*miR*s were of 111.4 bp length and mature *miR*s ranged from 18 to 24 (Fig. 3a) with mean of 20.73 (Table 3). Of predicted *miR*s, *miR*482 family had more members (six) representing its presence as one of the supreme molecule in the consortia of horsegram regulatory *miR*s. Similarly, *miR*156 and *miR*390 were found to have two members each whereas, other *miR*s have singlets from each family (Fig. 3b). Although, no *miR*s were identified from ESTs and SSH library validating them as coding sequences, the TSA sequences showed *miRNA* frequency of 1 in 1937 contigs (Table 4). The results of the predicted *miR*s and its targets in horsegram were statistically analyzed and summarized in Tables 3 and 4.

**Table 3:** Summary statistics for precursor *miRNA*s of *M. uniflorum*.

Parameter	Mean	Standard Deviation	Minimum	Maximum
LP	111.4	12.02	91	130
LM	20.733	1.437	18	24
AU%	52.232	6.86	39.24	60.38
GC%	45.718	25.82	39.62	58.19
A	31.6		19	38
U/T	32.31		58.1	19
G	26		18	38
C	23.8		17	36
MFE	− 31.06		− 37.3	− 59.6
AMFE	28.37		− 31.34	− 48.85
MFEI	0.752		0.57	1.03

LP, Length of the pre-cursor *miRNA*; LM, Length of the mature *miRNA.*

**Table 4:** Summary of the outcomes of the bioinformatics approach adopted to identify *miRNA*s in horsegram.

Database accessed	NCBI dbEST EBI
Candidate number of ESTs	1050
Candidate number of TSA	27,997
Total number of candidate sequences	29,047
Candidate number of *miRNA*s	8496
Number of contigs containing potential precursors	10
Number of microRNA families	8
Number of microRNAs predicted	15
Frequency of horsegram *miRNA*	1 *miRNA* per 1937(approx) sequences

### Evolutionary relationship with soybean

**Figure 4:**
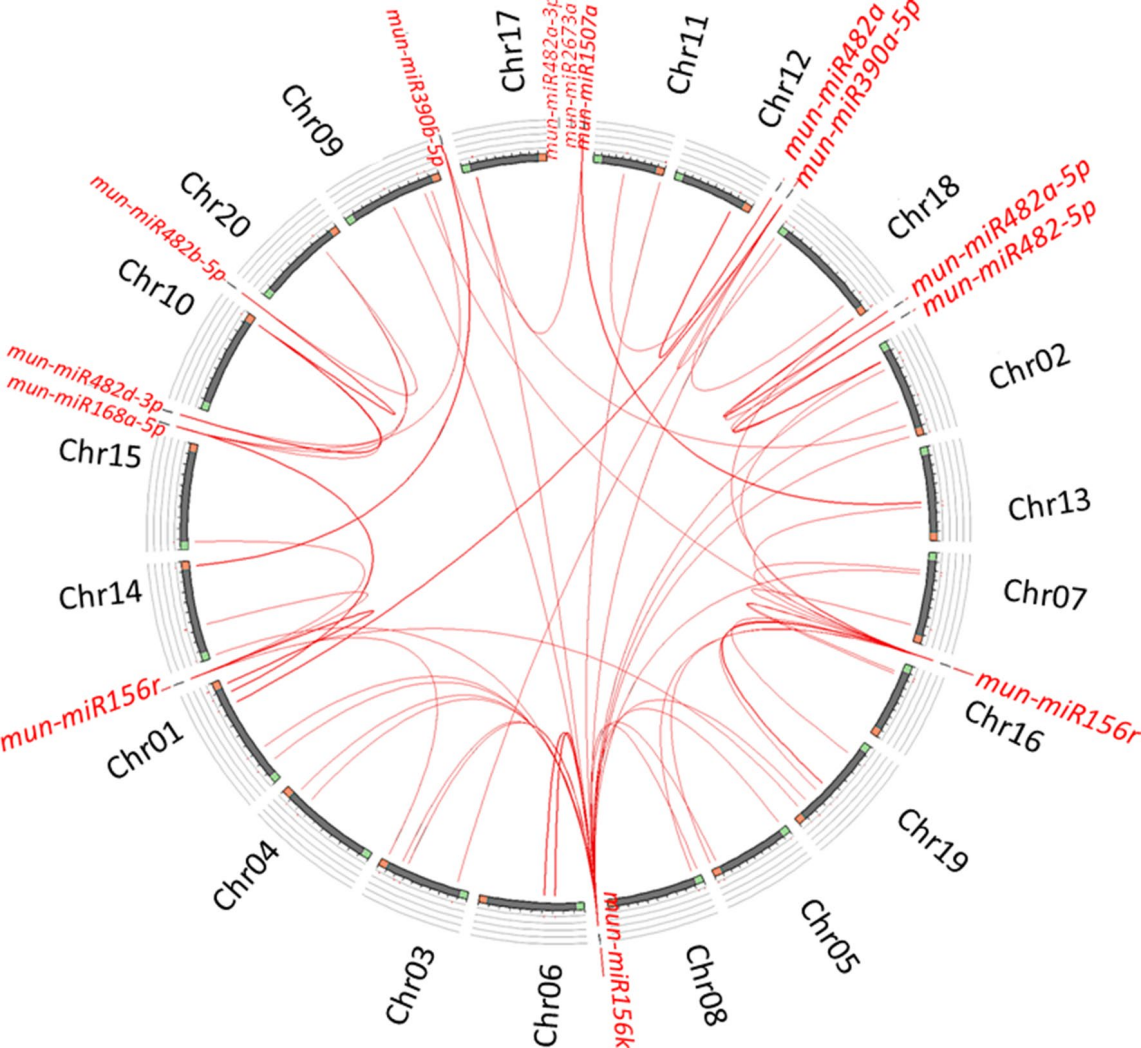
Synteny mapping of putative horsegram *miR*s with soybean genome. Synteny map explains the conserved sequences across species and their cross species transferability.

Soybean (*G. max* L.), the closely related model legume crop with complete genome information cracked has been used to establish a comparative syntenic map of predicted horsegram *miRNA*s to determine the putative regions of homologous *miR* genes. From mapping results, except mun-*miR*-482a-3p and *miR*2673a, rest of the putative horsegram *miRNA*s have orthologs widespread in *G. max* genome. *miR*156r and *miR*156k had more number of orthologs. The mapped *miR*s are depicted in soybean genome (Fig. 4). Comparative genomics enabled us to infer *miR* gene function, which could enumerate future research focus contributed by horsegram *miRNA*s in related species and in distant crops as well.

### Conservation and phylogenetic analysis

**Figure 5:**
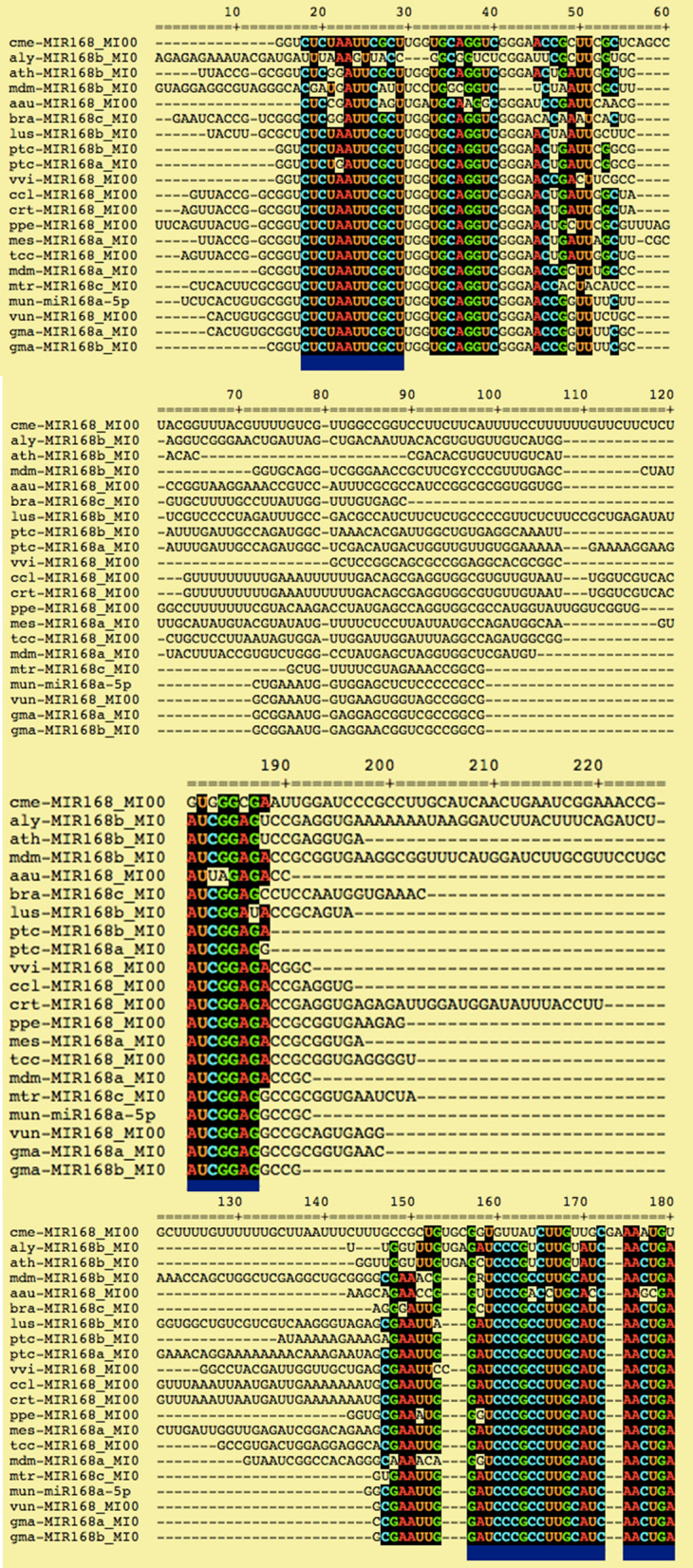
Pre-*miR* sequence conservation blocks for *miR*168 family of horsegram with related plants. Non-coding *RNAs* are conserved across species with specific role in development, metabolism and energy conservation.

**Figure 6:**
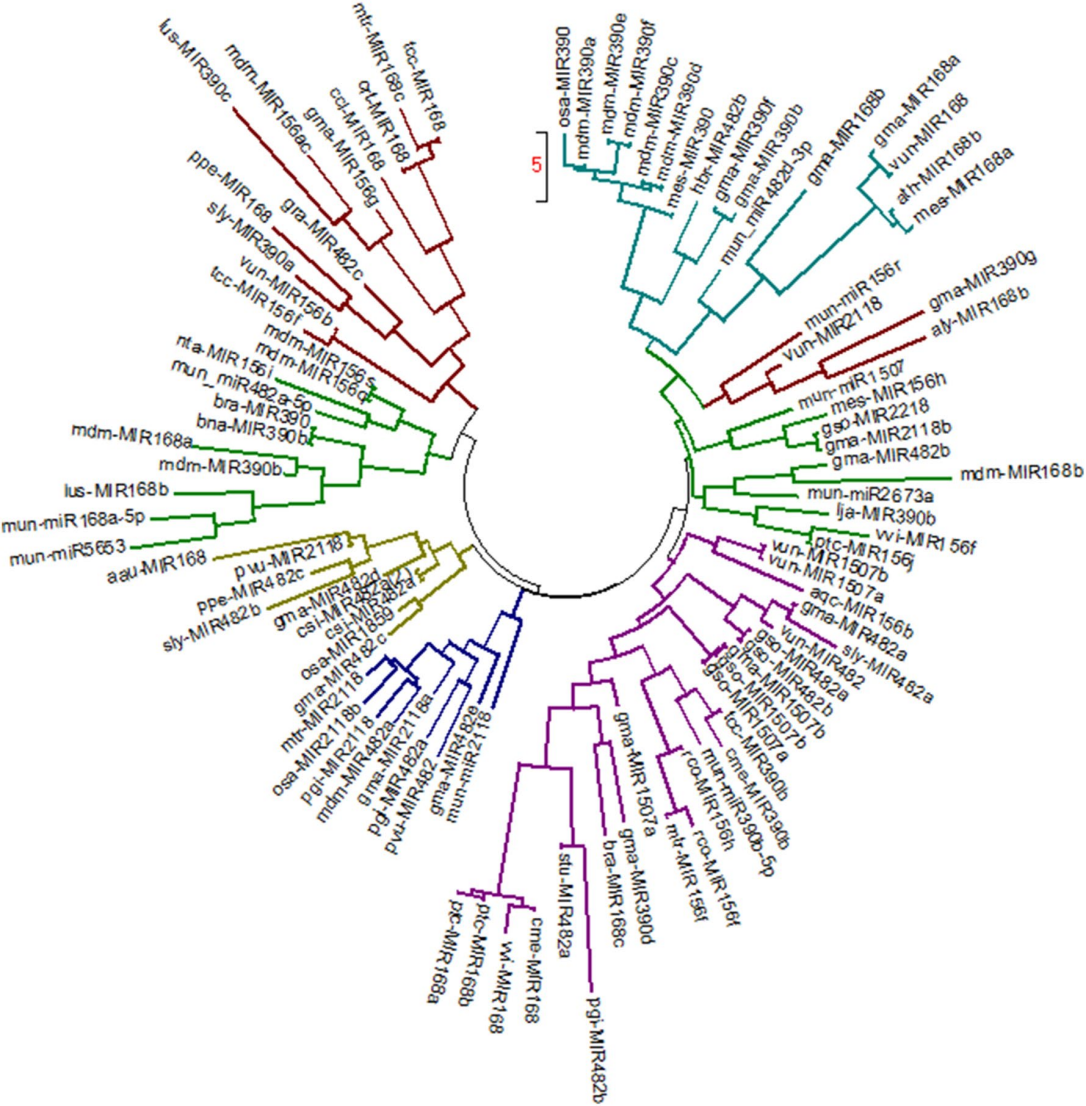
Phylogenetic analysis were done using MEGA7^30^ for predicted *miRNA* precursors of horsegram with their closely related plant *miR*s. Nine clusters of miR precursors clustered with related plant species.

**Figure 7:**
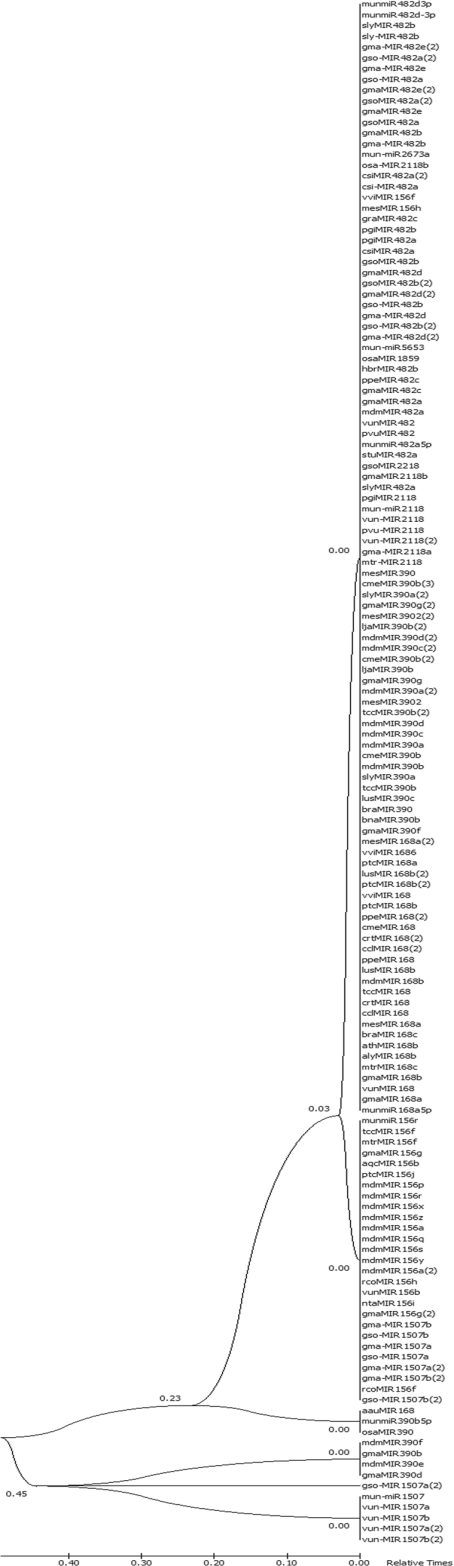
Phylogenetic tree of mun-*miR* families were constructed using PhyML^40^ and TreeDyn^41^. *miR* family 1507 is having related species *miR* in a separate cluster and their role in stress tolerance is also unique.

The pre-*miR* homologs were identified by performing blast of horsegram pre-*miRNA* against the *miR*Base database. The hits were filtered to retrieve only *miRNA*s of same family. In this study, high degree of conservation was exemplified by comparison of mun-*miR*168 to other plant precursor *miRNA*s (Figs. 5 and 6). It is evident that horsegram *miRNA*s share similarity with related legume species (Fig. 7). It is conclusively apparent that, horsegram *miRNA*s seem to have evolved at different rates in different time period similar to other plants.

### Putative target genes, their functions and networks

**Figure 8:**
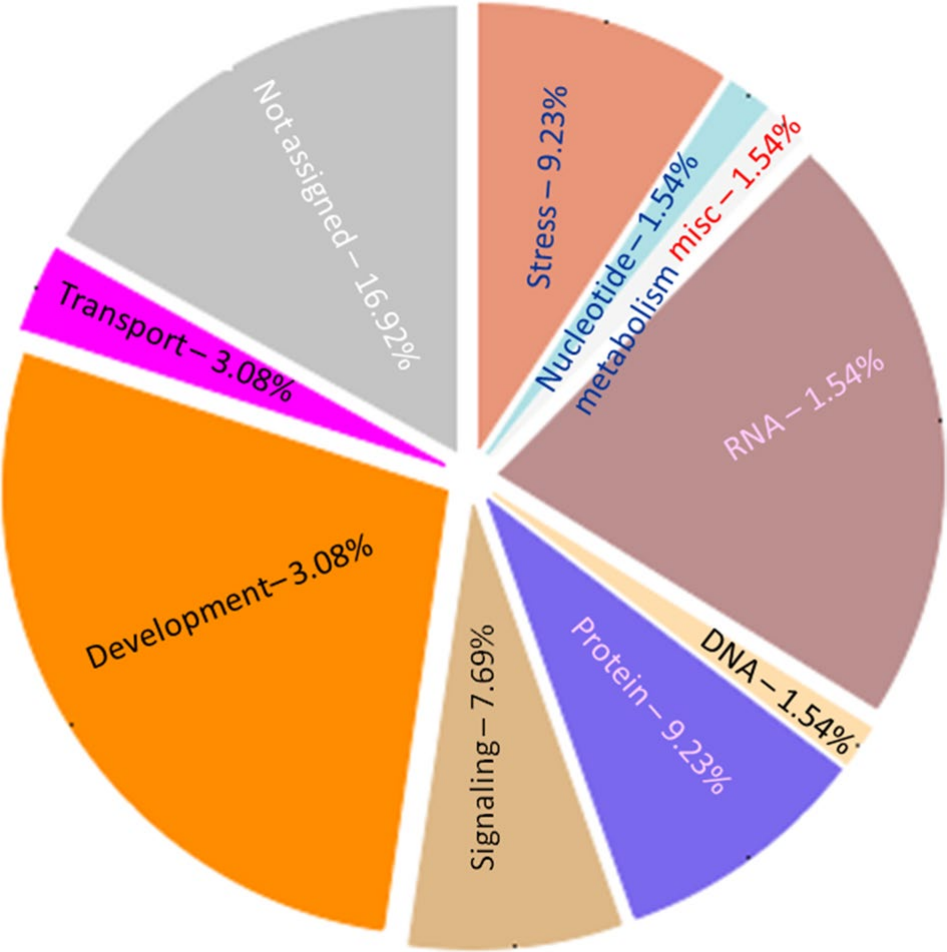
Mercator annotation results. Functional groups of identified transcripts based on annotation results drawn using Mercator^42^. Predicted *miR* targets ranging from RNA transcription regulation, protein post transcriptional modifications, development, signalling, biotic and abiotic stress tolerance and glycolysis were being regulated by the identified 15 *miR*s.

**Figure 9:**
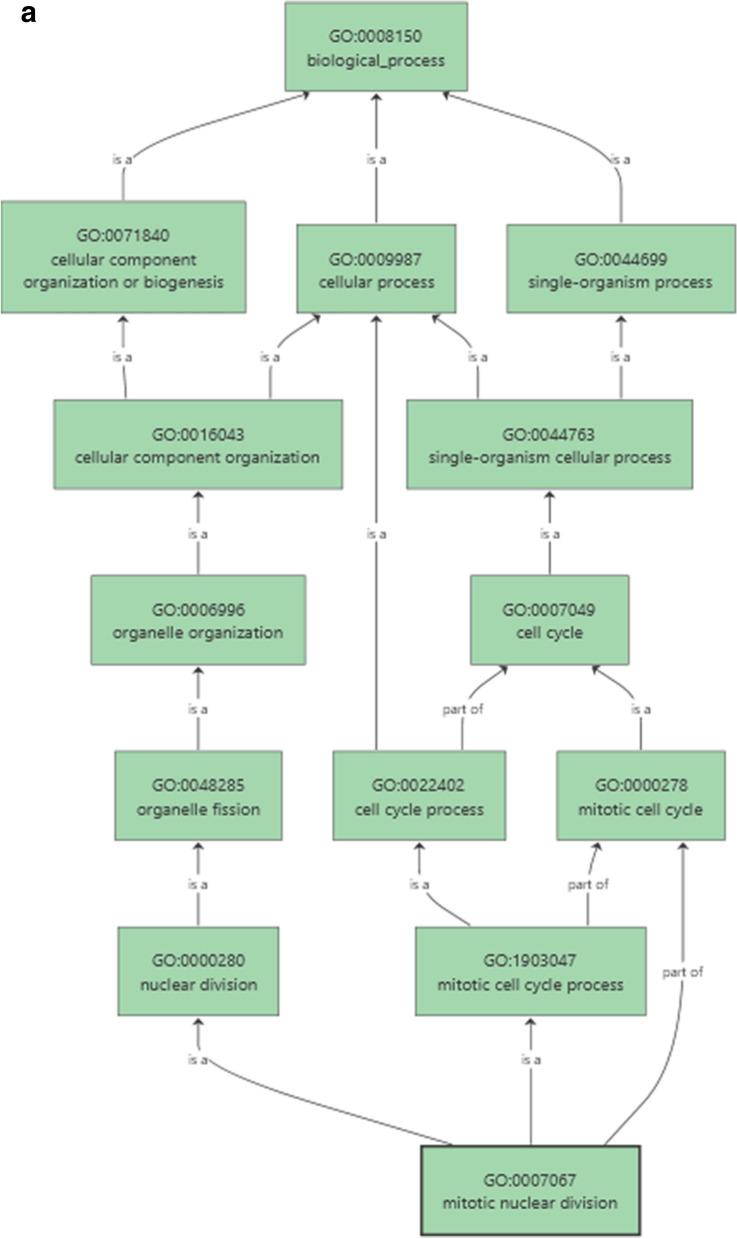

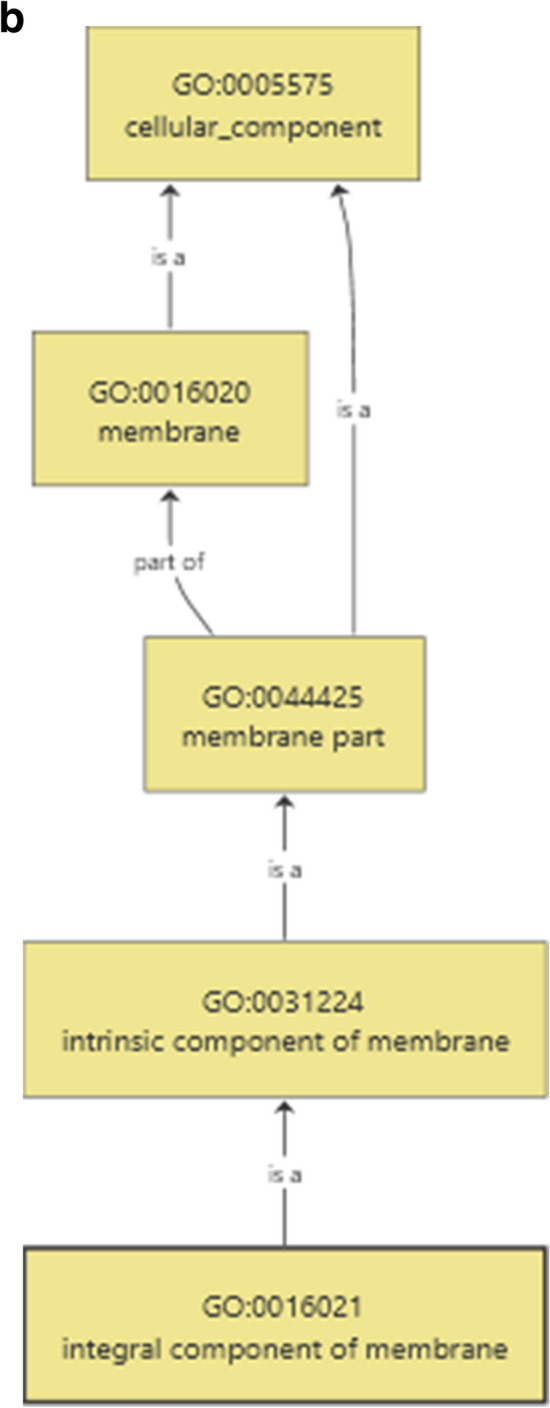
Target groups based on functional groups identification from B2g^44^. (**a**) Cell cycle, cell division and basic cell process were the key functions predicted among the *miR* target sequences. (**b**) Cell intergrity and intact cell membrane were being targeted indicating the activation of degradation pathways under stress conditions.

**Figure 10:**
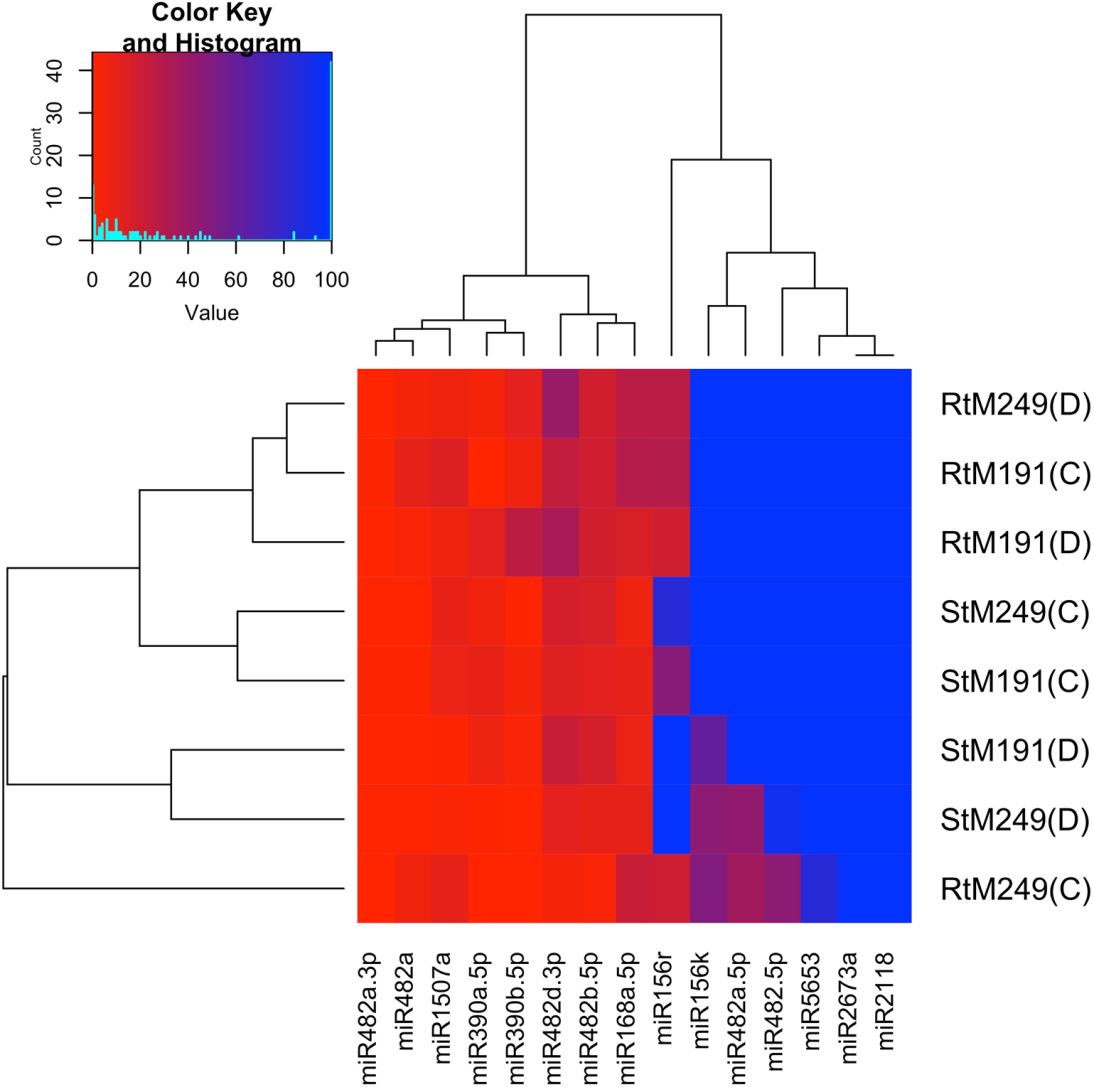
Expression heatmap of putative *miRNA*s differential expression in horsegram. Expression analysis clearly indicated two clusters of *miR*s. There is enough variation among the source tissue and gene families. The results of *in-silico* and real time quantitative expression pattern of *miR* are similar confirming prediction efficiency of the pipeline.

**Figure 11:**
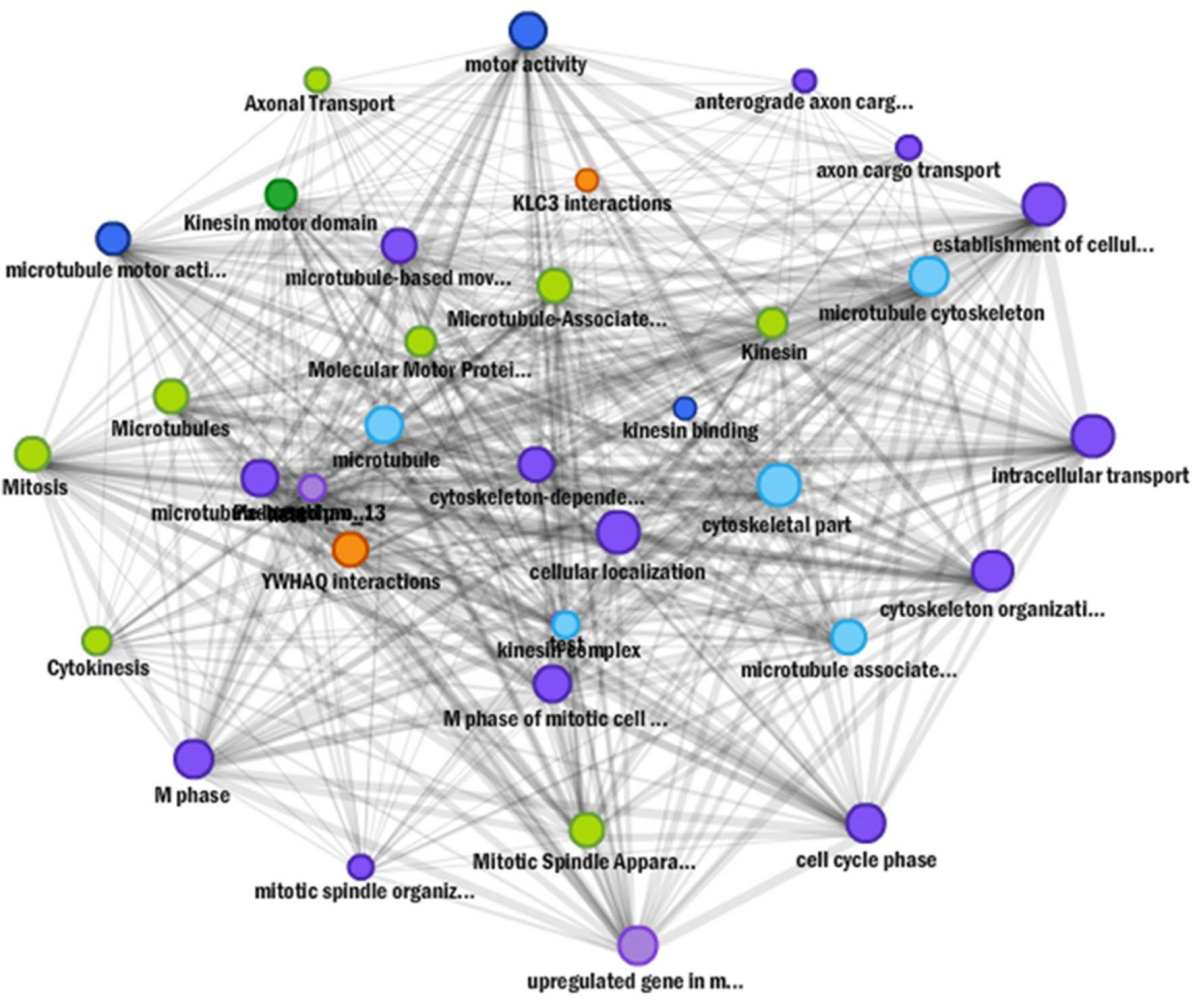
Predicted *miRNA* target genes function network. The function based protein target network drawn using sting server^82^ elucidates the role of *miR*s identified in cell development and maintenance of minimal functions during stress conditions. Most of the reductions in functions identified were correlated to energy conservation mechanism.

**Figure 12:**
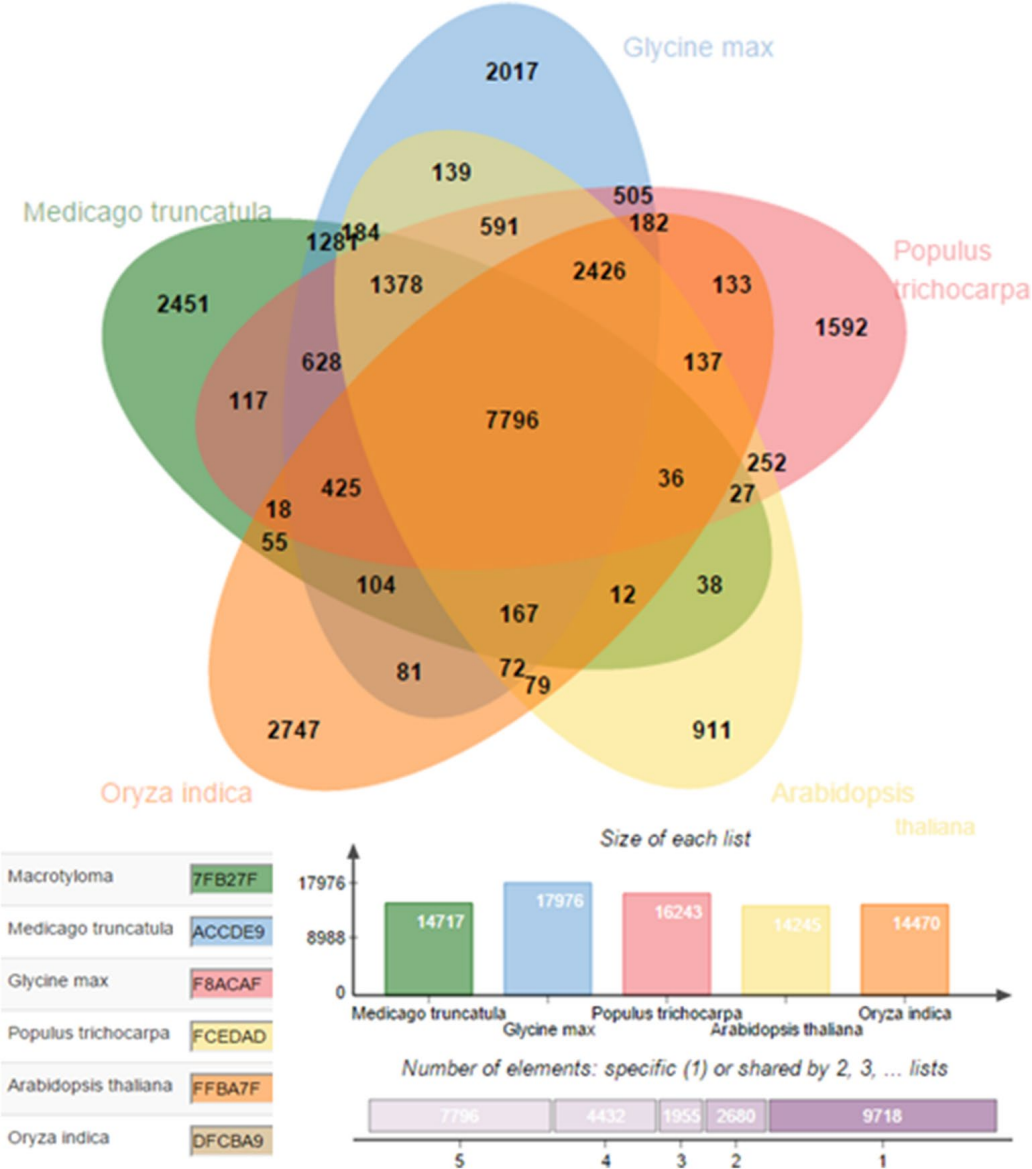
Predicted *miR* target whole genome wide homologs across species were identified using OrthoVenn2^83^. Like the conserved *miR*s across species, the target range is also conserved in different plant species indicating existence of similar mechanism.

Inferring the function of *miRNA* targets is crucial to significantly substantiate the functional role of *miRNA*s in gene expression and regulation. For predicted 15 horsegram *miR*s, 39 target genes were identified and classified into different groups based on their functional annotation. As revealed by Mercator annotation results (Fig. 8) of predicted *miR* targets, diverse processes ranging from RNA transcription regulation, protein posttranscriptional modifications, development, signalling, biotic and abiotic stress tolerance and glycolysis were being regulated by the identified 15 *miR*s. In addition, target genes also regulate cell wall degradation, hormone biosynthesis and redox components like ascorbate and glutathione synthesis (Table 5). The annotation of target genes using the Mercator tool categorized them into12 broad biochemical processes (Fig. 9). Differential expression of drought responsive *miRNA*s and gene networks Transcriptome analysis of Illumina sequence data from eight samples representing shoot and root tissues of contrasting horsegram genotypes M-191 (drought sensitive) and M- 249 (drought tolerant) (NCBI Bioproject PRJNA216977) was used to decipher the horsegram *miRNA* expression in root and shoot samples each under normal and drought stress condition respectively^11^. The *miRNA* abundance estimated from blast output indicates the behavioral *miRNA* expression (Fig. 10). The clustering simplifies the differential gene expression levels of horsegram *miRNA* and samples based on the similar expression. Network predicted and depicted as target- target interaction (Fig. 11) designates machineries of energy conservation confirming earlier hypothesis of structural compaction and energy conservation to survive under stress conditions^14,15^. This hypothesis may best fit and appropriate for other crops as well. To extend this hypothesis we identified target homologs in other plants also (Fig. 12).

**Table 5:** Functional annotation of horsegram *miRNA* targets.

*miRNA*_Acc	Target_Acc	Target_Description
mun-*miR*482d-3p	AT2G28380.1	DRB2/dsRNA-binding protein 2
	AT2G36470.1	Plant protein of unknown function (DUF868)
	AT4G06560.1	Transposable element gene
	AT3G30713.1	Transposable element gene
	AT5G65700.1	BAM1/Leucine-rich receptor-like protein kinase family protein
	AT5G65700.2	BAM1/Leucine-rich receptor-like protein kinase family protein
	AT4G06587.1	Transposable element gene
mun-*miR*482b-5p	AT1G78270.1	AtUGT85A4, UGT85A4/UDP-glucosyl transferase 85A4
	AT2G32700.5	LUH/LEUNIG_homolog
	AT2G32700.2	LUH/LEUNIG_homolog
	AT2G32700.4	LUH/LEUNIG_homolog
	AT2G32700.6	LUH/LEUNIG_homolog
	AT2G32700.1	LUH/LEUNIG_homolog
	AT2G32700.7	LUH/LEUNIG_homolog
	AT1G48550.1	Vacuolar protein sorting-associated protein 26
	AT1G48550.2	Vacuolar protein sorting-associated protein 26
	AT5G28650.1	WRKY74, ATWRKY74/WRKY DNA-binding protein 74
	AT3G58640.2	Mitogen activated protein kinase kinase kinase-related
	AT3G58640.1	Mitogen activated protein kinase kinase kinase-related
mun-*miR*482a-3p	AT1G72050.2	TFIIIA/transcription factor IIIA
	AT1G72050.1	TFIIIA/transcription factor IIIA
	AT4G03080.1	BSL1/BRI1 suppressor 1 (BSU1)-like 1
	AT5G12000.1	Protein kinase protein with adenine nucleotide alpha hydrolases-like domain
mun-*miR*482a-3p	AT1G22930.2	T-complex protein 11
mun-*miR*156r	AT2G16000.1	Transposable element gene
	AT3G45775.1	Transposable element gene
	AT2G43370.1	RNA-binding (RRM/RBD/RNP motifs) family protein
	AT5G50570.1	SPL13A, SPL13/Squamosa promoter-binding protein-like (SBP domain) transcription factor family protein
	AT5G50570.2	SPL13A, SPL13/Squamosa promoter-binding protein-like (SBP domain) transcription factor family protein
	AT2G42200.1	SPL9, AtSPL9/squamosa promoter binding protein-like 9
	AT5G50670.1	SPL13B, SPL13/Squamosa promoter-binding protein-like (SBP domain) transcription factor family protein
	AT3G57920.1	SPL15/squamosa promoter binding protein-like 15
	AT1G27370.1	SPL10/squamosa promoter binding protein-like 10
	AT1G69170.1	Squamosa promoter-binding protein-like (SBP domain) transcription factor family protein
	AT1G27370.2	SPL10/squamosa promoter binding protein-like 10
	AT5G43270.1	SPL2/squamosa promoter binding protein-like 2
	AT1G27370.4	SPL10/squamosa promoter binding protein-like 10
mun-*miR*1507a	AT2G15970.2	COR413-PM1 | cold regulated 413 plasma membrane 1
	AT4G10465.1	Heavy metal transport/detoxification superfamily protein
	AT2G15970.1	COR413-PM1, WCOR413, WCOR413-LIKE, ATCOR413-PM1, FL3-5A3, ATCYP19 |/cold regulated 413 plasma membrane 1
	AT4G06678.1	Transposable element gene
	AT5G35142.1	Transposable element gene
	AT5G44925.1	Transposable element gene
	AT3G21250.1	ATMRP6, MRP6, ABCC8 | multidrug resistance-associated protein 6 |
	AT3G21250.2	MRP6, ABCC8 | multidrug resistance-associated protein 6 |
	AT3G54510.2	Early-responsive to dehydration stress protein (ERD4)
	AT3G54510.1	Early-responsive to dehydration stress protein (ERD4)
mun-*miR*168a-5p	AT1G48410.3	AGO1/Stabilizer of iron transporter SufD/Polynucleotidyl transferase
	AT1G48410.1	AGO1/Stabilizer of iron transporter SufD/Polynucleotidyl transferase
	AT1G48410.2	AGO1/Stabilizer of iron transporter SufD/Polynucleotidyl transferase
mun-*miR*2118	AT2G21230.2	Basic-leucine zipper (bZIP) transcription factor family protein
	AT2G21230.1	Basic-leucine zipper (bZIP) transcription factor family protein
	AT2G21230.3	Basic-leucine zipper (bZIP) transcription factor family protein
	AT5G45050.2	TTR1, ATWRKY16, WRKY16/Disease resistance protein (TIR-NBS-LRR class)
	AT5G45050.1	TTR1, ATWRKY16, WRKY16/Disease resistance protein (TIR-NBS-LRR class)
	AT3G05680.1	EMB2016/embryo defective 2016
	AT3G05680.2	EMB2016/embryo defective 2016 |
	AT1G50120.1	Unknown function/Expressed
mun-*miR*482a	AT5G15850.1	COL1, ATCOL1/CONSTANS-like 1
	AT1G74600.1	Pentatricopeptide (PPR) repeat-containing protein
	AT5G15840.1	CO, FG/B-box type zinc finger protein with CCT domain
	AT5G15840.2	CO, FG/B-box type zinc finger protein with CCT domain
	AT2G21230.2	Basic-leucine zipper (bZIP) transcription factor family protein
	AT2G21230.1	Basic-leucine zipper (bZIP) transcription factor family protein
	AT2G21230.3	Basic-leucine zipper (bZIP) transcription factor family protein
	AT1G18300.1	atnudt4, NUDT4/nudix hydrolase homolog 4
mun-*miR*390b-5p	AT1G58050.1	RNA helicase family protein
	AT5G67610.1	Uncharacterized conserved protein (DUF2215)
	AT5G67610.2	Uncharacterized conserved protein (DUF2215)

## Discussion

**Figure 13:**
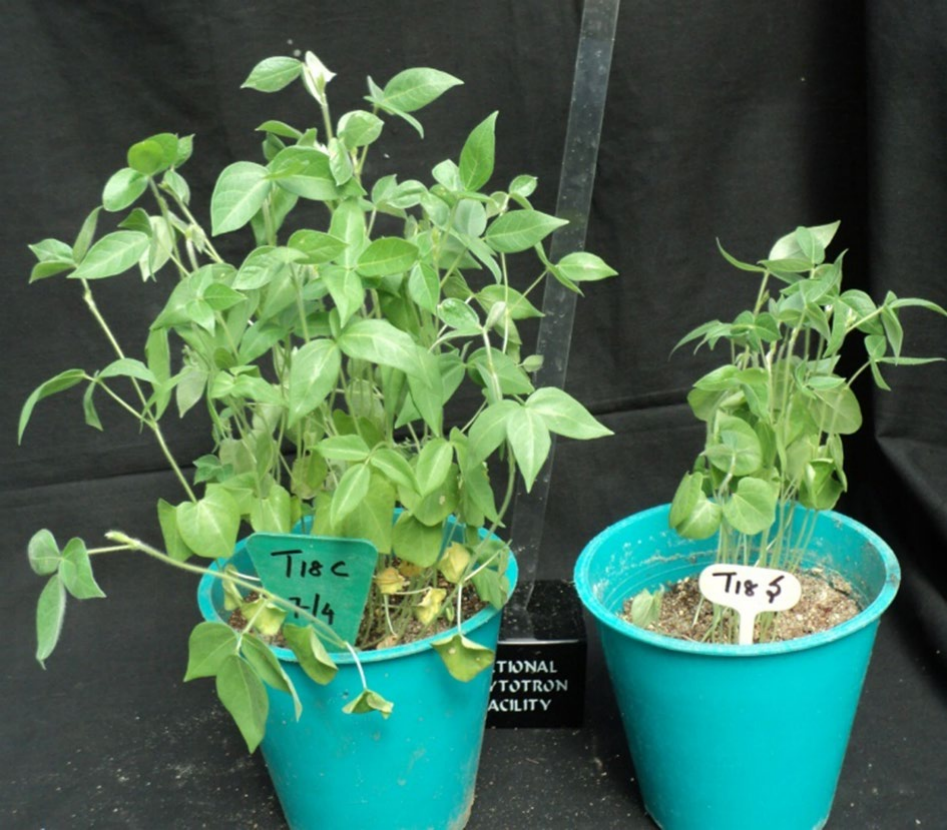
Control and stress treatments of horsegram plants. Paiyur1 plants were grown under glass house conditions. C. control plant pot was maintained with frequent recharging of soil moisture by irrigation; S. Plants grown under severe soil moisture deficit condition. Day time the plants suffered temporary wilting and regains during evening hours.

**Figure 14:**
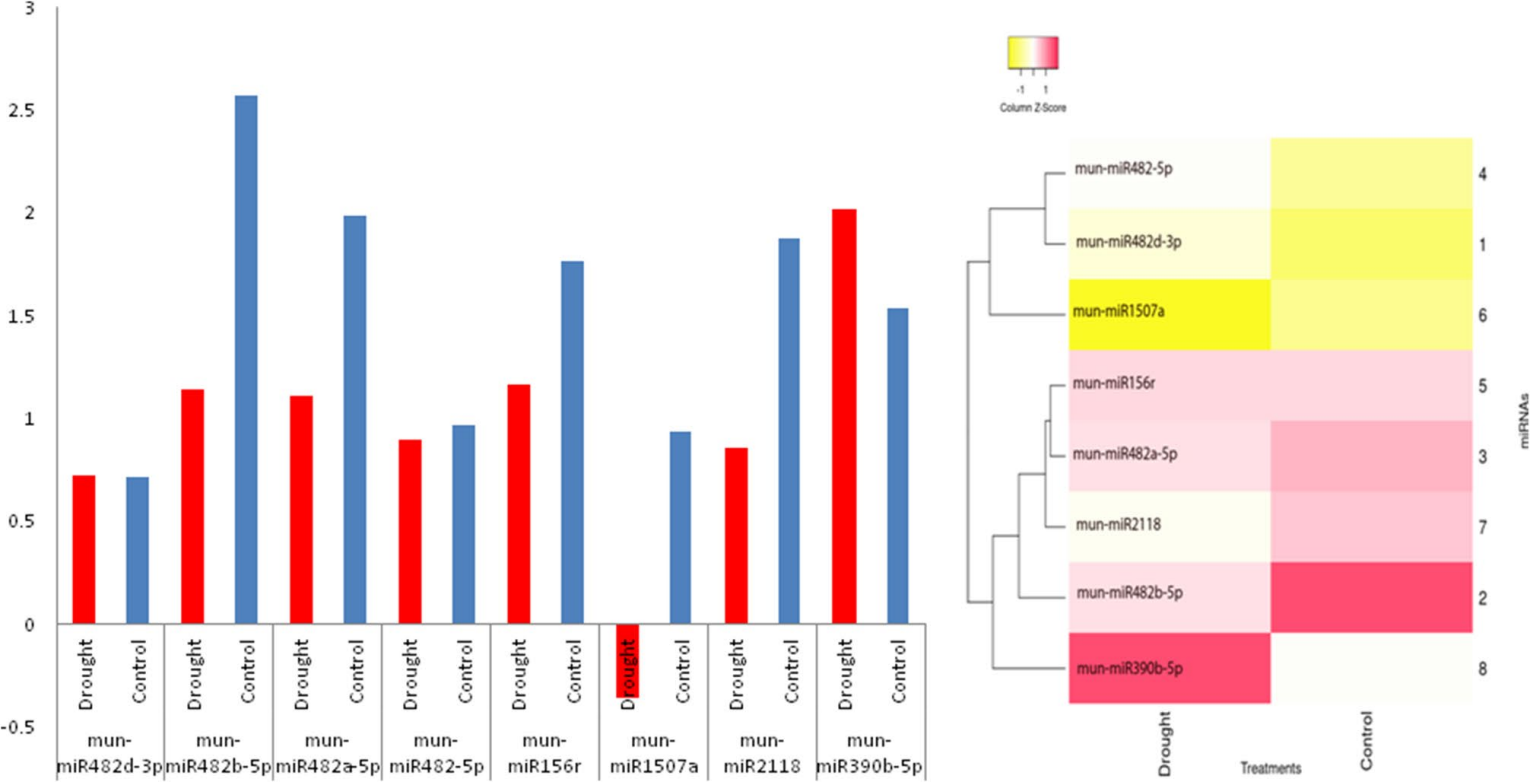
Quantitative rtPCR based differential expression of identified *mun-miR*s. Heatmaps were generated using Heatmapper^84^. Quantitative expression of *miR*s formed two clusters. Expression of *miR*1507 and *miR*390 families defines Paiyur1 phenotypic response to stress conditions. Down-regulation of other family *miR*s could be correlated to decline in cellular functions under stress conditions.

Comprehensive studies on plant *miRNA*s endorse stage specificity and multitude of targets^47–49^. The non-coding *miRNA*s play a regulatory role in its target protein coding gene expression^50^. The *miRNA* multiplexes with *RNA* induced silencing complex (RISC) guiding the repression or cleavage of its target messenger RNA by seed nuclei base-pairing^3^. Complementarity between *miRNA*s and their target genes are high to regulate developmental processes, metabolism and stress responses^51,52^. Hence, there is enormous necessity to identify and validate *miRNA*s for further downstream applications in plants^22^. In horsegram like deficit moisture stress tolerant crop, major genes interaction network influence in expression of a tolerant phenotype. From contrasting stress responsive genotype data, and qPCR of identified miRNAs from a single stress responsive genotype under irrigated and deficit stress (Fig. 13), we could identify differential expression of *miR* genes. These predicted 15 *miR*s were clustered in to 11 different families and are conserved. To validated identified miRNAs qPCR was performed as reported earlier (53). Of the total eight miRs validated, two clusters were formed. Mun-miR 482 was found in both the clusters; whereas, mun-miR1507 was found in one cluster which distinguish the tested variety Paiyur1 for its ability to react for stress (Fig. 14). Of the 15 identified mun-miRs, miR 156, miR 171 and miR 390 were found to be differentially expressed in germinating seeds of halophyte *Reaumuria soongorica* under salt stress conditions (54). Legume genomes are rich in SNPs. Saturated map of SNPs were reported in legumes like vigna (55) and In pigeonpea (56). Of which highest haplotype desnsity of 0.7380 was reported for serine threonine kinase coding disease resistance gene (56) which was identified as an important miR target in the present ivestigation. Variations in these miR sequences are expected among germplasm to change their expression. This could be a reason for non expression of other genes. The conserved nature of maximum plant *miRNA*s bolstered the *miRNA* search in different plant species utilizing available genomic resources like ESTs, GSS and *mRNA* sequences^34,57–59^ to a great extent and we followed the trend in identifying them in a drought tolerant crop. Conserved *miRNA*s in ginger, garlic, coffee and tea were identified^60–63^. For predicted pre-*miRNA*s, MFEI resolution for length variation extends from 0.57 to 1.03 in horsegram. Pre-*miR*s illustrate the absence of large internal loops/bulges and at least 20 nucleotides for Wobble base pairing (G/U base pairings) or Watson–Crick base pairing between the *miRNA* and the star sequence^62,64^. Estimated (A + U) % of pre-*miR*s range satisfies the predetermined criteria^18^. The putative genomic region of these non-coding sequences were determined and intergenic sequences were utilized for secondary structure prediction with the stringent filtering criteria to attain potential precursor *miRNA*^26^. Resultant secondary structures were inspected for precursor *miRNA* and the positioning of the mature *miRNA* within its stem-loop structure manually. The sequences with suitable secondary structure^66^ were characterized for structure attributes and its possible existence as potential precursor of the predicted *miR*s. The transcription of *miRNA*s from sense and antisense strands of genes were already reported^67^. Our results stand by the possibility of the *miRNA* in both sense and antisense strands (Table 2) similar results were already reported in potato, tobacco and B. rapa^16,68,69^. The frequency of *miRNA* using expressed sequence tags is 1 in 1000 confirming previous reports^62^. The interaction of *miRNA*s with its target genes can enumerate evolutionary role of microRNAs^48,70,71^. Utilization of *miRNA*s in RNA interference (RNAi) mediated gene regulation has emerged as an important tool in novel traits engineering either by over expression of a *miRNA* or target genes by synthetic *miRNA*s. Gene silencing/knockout may help in understanding *miRNA* in plant responses to stress resulting increased productivity with improved nutritional value^72^. Cellular functions were not known for eight of the target proteins identified in the present investigation. Biotic/Abiotic stresses are critically affecting growth and development of plants. There are reports on contrasting mechanisms confirming grades of deficit moisture stress condition to control among different horsegram germplasm sources^6,15^. Among the identified *miRNA*s mun-*miR*156r, mun-*miR*156k, mun-*miR*482a, mun-*miR*390b-5p and mun-*miR*482a-5p were noticed to be involved in RNA processing, protein synthesis and modifications as well as plant development by altering the expression of their respective targets, whereas the genes encoding abiotic stress, biotic stress (NBS-LLR resistant class) and signalling associated proteins were targeted by mun-*miR*482a-5p, mun-*miR*482d-3p and mun-*miR*1507a respectively. Thus, there is concurrent substantiation that microRNAs can play pivotal role in crop improvement^73^ and the *miRNA*s predicted from this investigation can be useful for future research. Closely related homologs of predicted putative *miR*s exhibited high degree of conservation as in mum-*miR*482 with related plant mature *miRNA*s (Fig. 6). The precursor sequences from *miR*482, *miR*390 and *miR*150 represented a strong candidate promulgating its necessary importance at post-transcriptional gene regulation in horsegram. The conserved nature of precursor and mature *miRNA*s has been reported in various plant groups from earlier studies^16,34,73,74^. The potential of *miRNA*s to bind corresponding target *mRNA* are comparable with their complementarity to degrade target *mRNA*. Therefore, to infer the contribution of microRNAs in cellular functions and regulatory gene networks, the *miRNA* target gene prediction is a crucial step^64^. The majority of the predicted *miRNA* targets in horsegram depict energy conservation mechanism which play important role in survival during adverse conditions. Target gene prediction reveals the regulation of multiple genes by single *miRNA* with different levels of regulation^22,47,75^. Also, the genes targeted belong to more than one gene family, which shows the multitude of *miRNA* functions in various metabolic processes (Table 4). We could spot 39 target genes of predicted *miR*s in horsegram and its homologs in other plant species. This indicates that, drought tolerance phenotype can be manipulated by adjusting the expression of identified *miR*s and their targets.

## Conclusions

**Figure 15:**
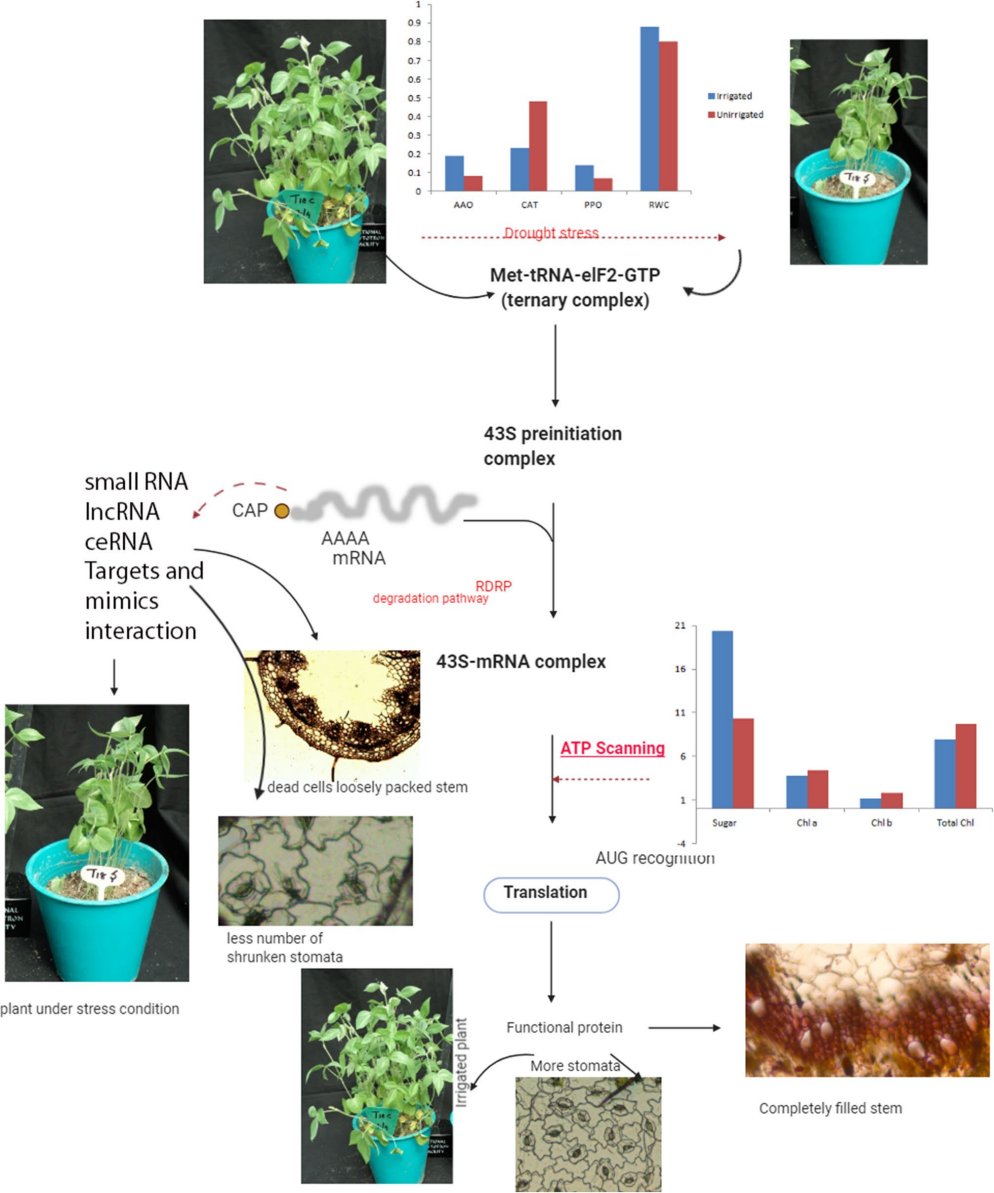
Control and stress treatments of horsegram plants and perspective conclusion of the predicted stress tolerance mechanism. Illustration was drawn with wet lab results and glass house grown plant sample analyses. Under stress conditions AAO expression defines the tolerance with declined total sugar and chlorophyll contents. Pith autolysis and shrunken stomata was observed under soil moisture deficit stress.

Great concern is being publicized recently to unravel various mechanisms involved in drought tolerance with emphasis to changing climatic conditions. Even though, there are extensive inquiries on *miRNA*s discovery and their functional prediction were completed, some of the non-model plants with considerable traits were not subjected to systematic investigation to elucidate contrasting mechanisms and their key players. Eight novel *miRNA*s were^76^ predicted from horsegram. However, these non-validated *miRNA*s were predicted with low stringency and standard nomenclature was not followed. Inappropriately, the drought hardy horsegram *miRNA*s still remain unknown and lacks extensive validation. In this study, 15 conserved *miRNA*s belonging to nine different families were identified from EST and TSA sequences and validated in this first report with its tissue specific differential expression (Fig. 10). Mun-*miR*482a-5p and mun-*miR*482d-3p are *miR*482 family *miRNA*s reported to regulate stress response in soybean and apple^77,78^. The same trend is observed in our results confirming the earlier report. Mun-*miR*390b-5p is a *miR*390b family *miRNA* earlier found for protein degradation and post transcriptional modifications in Arabidopsis, maize and soybean^79–81^. Upregulation of *miR*390b and downregulation of 1507 family *miRNA* was observed (Fig. 14) in qPCR of Paiyur1 variety which is a stress responsive accession. The present investigation indirectly links stress tolerance to energy conservation as indicated by the network rather than direct response to stress conditions (Fig. 15). Hence, we confirm the interplay of *miRNA*-stress response-NBS-LLR class R-protein response^77^ in energy conservation to survive the stress conditions. Additionally, the study reveals that identified *miRNA* regulated target genes have differential biological functions including cell wall degradation, hormone synthesis and synthesis of redox component like ascorbate and glutathione (Table 5). These findings can further help in translational research of related legumes and may result in selection of better germplasm for higher productivity and nutritional security. The network predicted from this investigation confirms the earlier hypotheses: structural compaction to overcome stress tolerance^14,15^. Altogether, the outcomes of the present investigation deliver clarity that, horsegram is a drought adapted crop and can be considered as a model crop for drought tolerance research. Thus, the predicted horsegram *miRNA*s may unravel the unique tolerance capability associated to its metabolic pathways and the present workflow represents simple and straightforward approach for the prediction and characterization of *miRNA*s in those plants for which genomes are yet to be splintered.

## Supplementary information

Supplementary Table.

## References

[1] Jones-Rhoades MW, Bartel DP, Bartel B (2006). MicroRNAs and their regulatory roles in plants. Annu. Rev. Plant Biol..

[2] Pritchard CC, Cheng HH, Tewari M (2012). MicroRNA profiling: approaches and considerations. Nat. Rev. Genet..

[3] Bartel DP (2004). MicroRNAs: genomics, biogenesis, mechanism, and function. Cell.

[4] Arora RK, Chandel KPS (1972). Botanical source areas of wild herbage legumes in India. Trop. Grasslands..

[5] Yasin, J. K. *et al*. Identification and validation of genes responsible for moisture stress tolerance in Horsegram (*Macrotyloma uniflorum*(Lam.) Verdc.), LIBEST_027585.2011. https://www.ncbi.nlm.nih.gov/biosample/SAMN00750282 (2011).

[6] Yasin JK, Bhat KV, Nizar MA, Rajkumar S, Verma M, Radhamani J, Verma N, Pandey S (2014). Alternate antioxidant defence system in moisture stress responsive accessions of horse gram. Legume Res..

[7] Cook, B.G., Pengelly, B.C., Brown, S.D., Donnelly, J.L., Eagles, D.A., Franco, M.A., Hanson, J., Mullen, B.F., Partridge, I.J. & Peters, M. Tropical Forages: An interactive selection tool (2005).

[8] Morris JB (2008). Macrotyloma axillare and M. uniflorum: descriptor analysis, anthocyanin indexes, and potential uses. Genet. Resour. Crop Evol..

[9] Peshin A, Singla SK (1994). Anticalcifying properties of *Dolichos biflorus*(horse gram) Seeds. Indian J. Exp. Biol..

[10] Reddy PCO, Sairanganayakulu G, Thippeswamy M, Reddy PS, Reddy MK, Sudhakar C (2008). Identification of stress-induced genes from the drought tolerant semi-arid legume crop horsegram (*Macrotyloma uniflorum* (Lam.) Verdc.) through analysis of subtracted expressed sequence tags. Plant Sci..

[11] Bhardwaj J, Chauhan R, Swarnkar MK, Chahota RK, Singh AK, Shankar R, Yadav SK (2013). Comprehensive transcriptomic study on horse gram (*Macrotyloma uniflorum*): De novo assembly, functional characterization and comparative analysis in relation to drought stress. BMC Genomics.

[12] Yasin, J.K., Mishra, B. K., Chaudhary, S., Magadum, S., Chinnusamy, V. & Singh, N.K. Transcriptome analyses for genome wide identification of ncRNAs and *miRNA* from Pigeonpea (*Cajanus cajan*L.). In “Plant Biology 2016” 13–18th July, 2016 by ASPB, Austin, Texas, USA (2016).

[13] Yasin, J.K., Sreevathsa, R., Vivek, T., Nager, R., Lal, S.K., Pillai, M.A. & Chinnusamy, V. lncRNA plays a major role in Susceptibility to resistance: Insights into pigeonpea RGA. In “Plant Biology 2016” 13–18th July, 2016 by ASPB, Austin, Texas, USA (2016).

[14] Yasin, J.K. & Magadum, S. Structural compaction to conserve energy: ncRNA expression directs pH flux of floral parts and yield loss in pigeonpea (*Cajanus cajan*L.). In Royal Society Theo murphy meeting on “Evolution brings Ca2+ and ATP together to control life and death. March 16–17th 2016 at Royal Society of UK, London (2016).

[15] Yasin, J.K., Bhat, K.V., Rajkumar, S., Subalakshmi, Ramya, K.T. & Fiyaz, A.R. Structural compaction, Mechanism of acid tolerance in moisture stress responsive accessions of horse gram. In The 8TH International symposium on “Plant soil interactions at low pH”, October 18-22, 2012, Bangalore, India (2012); 170

[16] Dhandapani V, Ramchiary N, Paul P, Kim J, Choi SH, Lee J, Hur Y, Lim YP (2011). Identification of potential microRNAs and their targets in *Brassica rapa* L. Mol. Cells..

[17] Xie FL, Huang SQ, Guo K, Xiang AL, Zhu YY, Nie L, Yang ZM (2007). Computational identification of novel microRNAs and targets in *Brassica napus*. FEBS Lett..

[18] Zhang BH, Pan X, Cobb GP, Anderson TA (2006). Evidence that *miRNA*s are different from other RNAs. Cell Mol. Life Sci..

[19] Yang T, Xue L, An L (2007). Functional diversity of *miRNA* in plants. Plant Sci..

[20] Gupta PK (2015). MicroRNAs and target mimics for crop improvement. Curr. Sci..

[21] Zhang BH, Pan XP, Wang QL, Cobb GP, Anderson TA (2005). Identification and characterization of new plant microRNAs using EST analysis. Cell Res..

[22] Zhang BH, Pan X, Cobb GP, Anderson TA (2006). Plant microRNA: a small regulatory molecule with big impact. Dev. Biol..

[23] Jones GS, Grocock RJ, Van Dongen S, Bateman A, Enright AJ (2006). *MiR*Base: microRNA sequences, targets and gene nomenclature. Nucl. Acids Res..

[24] Li W, Godzik A (2006). Cd-hit: a fast program for clustering and comparing large sets of protein or nucleotide sequences. Bioinformatics.

[25] Altschul SF, Gish W, Miller W, Myers EW, Lipman DJ (1990). Basic local alignment search tool. J. Mol. Biol..

[26] Zuker M (2003). Mfold web server for nucleic acid folding and hybridization prediction. Nucl. Acids Res..

[27] Dai X, Zhao PX (2011). psRNATarget: a plant small RNA target analysis server. Nucl. Acids Res..

[28] Darzentas N (2010). Circoletto visualizing sequence similarity with Circos. Bioinformatics.

[29] Krzywinski M, Schein J, Birol I, Connors J, Gascoyne R, Horsman D, Jones S, Marra MA (2009). Circos: an information aesthetic for comparative genomics. Genome Res..

[30] Kumar S, Stecher G, Tamura K (2016). MEGA7: Molecular Evolutionary Genetics Analysis version 7.0 for bigger datasets. Mol. Biol. Evol..

[31] Kong L, Zhang Y, Ye ZQ, Liu XQ, Zhao SQ, Wei L, Gao G (2007). CPC, assess the protein coding potential of transcripts using sequence features and support vector machine. Nucl. Acids Res..

[32] Yi X, Zhang Z, Ling Y, Xu W, Su Z (2015). PNRD: a plant non-coding RNA database. Nucl. Acids Res..

[33] Singh J, Nagaraju J (2008). In silico prediction and characterization of microRNAs from red flour beetle (*Tribolium castaneum*). Insect Mol. Biol..

[34] Zhang BH, Pan XP, Cox SB, Cobb GP, Anderson TA (2006). Evidence that *miRNA*s are different from other RNAs. Cell. Mol. Life Sci..

[35] Wang J, Hou X, Yang X (2011). Identification of conserved microRNAs and their targets in Chinese cabbage (*Brassica rapa* subsp. pekinensis). Genome..

[36] Ambros V, Bartel B, Bartel DP (2003). A uniform system for microRNA annotation. RNA.

[37] Dereeper V, Guignon G, Blanc S, Audic S, Buffet F, Chevenet JF, Dufayard S, Guindon V, Lefort M, Lescot M (2008). Phylogeny. Fr: robust phylogenetic analysis for the non-specialist. Nucl. Acids Res..

[38] Edgar RC (2004). MUSCLE: multiple sequence alignment with high accuracy and high throughput. Nucl. Acids Res..

[39] Castresana J (2000). Selection of conserved blocks from multiple alignments for their use in phylogenetic analysis. Mol. Biol. Evol..

[40] Guindon S, Gascuel O (2003). A simple, fast, and accurate algorithm to estimate large phylogenies by maximum likelihood. Syst. Biol..

[41] Chevenet F, Brun C, Banuls AL, Jacq B, Chisten R (2006). TreeDyn: towards dynamic graphics and annotations for analyses of trees. BMC Bioinform..

[42] Lohse M, Nagel A, Herter T, May P, Schroda M, Zrenner R, Tohge T, Fernie AR, Stitt M, Usadel B (2014). Mercator: a fast and simple web server for genome scale functional annotation of plant sequence data. Plant Cell Environ..

[43] Tian T, Liu Y, Yan H, You Q, Yi X, Du Z, Su Z (2017). agriGO v2.0: a GO analysis toolkit for the agricultural community, 2017 update. Nucl. Acids Res..

[44] Conesa A, Götz S (2008). Blast2GO: A comprehensive suite for functional analysis in plant genomics. Int. J. Plant Genomics..

[45] Bonnet E, Wuyts J, Rouze P, Van de Peer Y (2004). Detection of 91 potential conserved plant microRNAs in Arabidopsis thaliana and *Oryza sativa*identifies important target genes. Proc. Natl. Acad. Sci. USA.

[46] Bonnet E, Wuyts J, Rouze P, Van de Peer Y (2004). Evidence that microRNA precursors, unlike other non-coding RNAs, have lower folding free energies than random sequences. Bioinformatics.

[47] Yin VP, Thomson JM, Thummel R, Hyde DR, Hammond SM, Poss KD (2008). Fgf dependent depletion of microRNA-133 promotes appendage regeneration in zebrafish. Genes Dev..

[48] Sunkar R, Li YF, Jagadeeswaran G (2012). Functions of microRNAs in plant stress responses. Trends Plant Sci..

[49] Zhang B (2015). MicroRNA: a new target for improving plant tolerance to abiotic stress. J. Exp. Bot..

[50] Franco NB, Iniguez LP, Valdes-Lopez O, Alvarado X, Leija A, Fuentes S, RamiRez M, Paul S, Reyes J, Girard L (2015). The micro-RNA172c-APETALA2-1 node as a key regulator of the common bean-rhizobium *etli* Nitrogen fixation symbiosis. Plant Physiol..

[51] Kim VN (2005). MicroRNA biogenesis: coordinated cropping and dicing. Nat. Rev. Mol. Cell Biol..

[52] Chen X (2005). MicroRNA biogenesis and function in plants. FEBS Lett..

[53] Kang K, Zhang X, Liu H, Wang Z, Zhong J, Huang Z, Peng X, Zeng Y, Wang Y, Yang Y, Luo J (2012). A novel real-time PCR assay of microRNAs using S-Poly (T), a specific oligo (dT) reverse transcription primer with excellent sensitivity and specificity. PLoS ONE.

[54] Zhang H, Liu X, Yang X, Wu H, Zhu J, Zhang H (2020). miRNA–mRNA integrated analysis reveals roles for miRNAs in a typical halophyte, *Reaumuria soongorica*, during seed germination under salt stress. Plants.

[55] Yasin, J.K. High density SSR and SNP saturated physical maps of *Vigna radiata.* https://legumeinfo.org/genomes/gbrowse/Vr1.0 (2018).

[56] Singh S, Mahato AK, Jayaswal PK (2020). A 62K genic-SNP chip array for genetic studies and breeding applications in pigeonpea (*Cajanus cajan* L. Millsp.). Sci. Rep..

[57] Rogers K, Chen X (2013). Biogenesis, turnover, and mode of action of plant microRNAs. Plant Cell..

[58] Dezulian T, Palatnik JF, Huson D, Weigel D (2005). Conservation and divergence of microRNA families in plants. Genome Biol..

[59] Dezulian T, Schaefer M, Wiese R, Weigel D, Huson DH (2006). CrossLink: visualization and exploration of sequence relationships between (micro) RNAs. Nucl. Acids Res..

[60] Weber MJ (2005). New human and mouse microRNA genes found by homology search. FEBS J..

[61] Singh N, Srivastava S, Sharma A (2016). Identification and analysis of *miRNA*s and their targets in ginger using bioinformatics approach. Gene.

[62] Panda D, Dehury B, Sahu J, Barooah M, Sen P, Modi MK (2014). Computational identification and characterization of conserved miRNAs and their target genes in garlic (*Allium sativum* L.) expressed sequence tags. Gene..

[63] Akter MM, Islam SI, Mondal Z, Mahmud NA, Jewel S, Ferdous MR, Amin M, Rahman M (2014). Computational identification of *miRNA* and targets from expressed sequence tags of coffee (*Coffea arabica*). Saudi J. Biol. Sci..

[64] Das A, Mondal TK (2010). Computational identification of conserved microRNAs and their targets in tea (*Camellia sinensis*). Am. J. Plant Sci..

[65] Zhang BH, Pan XP, Cox SB, Cobb GP, Anderson TA (2006). Conservation and divergence of plant microRNA genes. Plant J..

[66] Wang L, Liu H, Li D, Chen H (2011). Identification and characterization of maize microRNAs involved in the very early stage of seed germination. BMC Genomics..

[67] Stark KL, Xu B, Bagchi A, Lai WS, Liu H, Hsu R, Wan X, Pavlidis P, Mills AA, Karayiorgou M (2008). Altered brain microRNA biogenesis contributes to phenotypic deficits in a 22q11-deletion mouse model. Nat. Genet..

[68] Xie YF, Shu R, Jiang SY, Liu DL, Zhang XL (2011). Comparison of microRNA profiles of human periodontal diseased and healthy gingival tissues. Int. J. Oral Sci..

[69] Frazier TP, Xie F, Freistaedter A, Burklew CE, Zhang B (2010). Identification and characterization of microRNAs and their target genes in tobacco (*Nicotiana tabacum*). Planta.

[70] Jha A, Shankar R (2011). Employing machine learning for reliable *miRNA* target identification in plants. BMC Genomics.

[71] Ding J, Li D, Ohler U, Guan J, Zhou S (2012). Genome-wide search for *miRNA*-target interactions in Arabidopsis thaliana with an integrated approach. BMC Genomics.

[72] Kamthan A, Chaudhuri A, Kamthan M, Datta A (2015). Small RNAs in plants: recent development and application for crop improvement. Front. Plant Sci..

[73] Li C, Zhang B (2016). MicroRNAs in control of plant development. J. Cell. Physiol..

[74] Catalano D, Pignone D, Sonnante G, Finetti-Sialer MM (2012). *In-silico* and in-vivo analyses of EST databases unveil conserved *miRNA*s from *Carthamus tinctorius*and *Cynara cardunculus*. BMC Bioinform..

[75] Rhoades MWJ, Bartel DP (2004). Computational identification of plant microRNAs and their targets, including a stress- induced *miRNA*. Mol. Cell..

[76] Bhardwaj S, Singh A, Singh P (2010). MicroRNA-based cancer therapeutics: big hope from small RNAs. Mol. Cell. Pharmacol..

[77] Zhai J, Jeong DH, De Paoli E, Park S, Rosen BD, Li Y, González AJ, Yan Z, Kitto SL, Grusak MA (2011). MicroRNAs as master regulators of the plant NB-LRR defense gene family via the production of phased, trans-acting siRNAs. Genes Dev..

[78] Xia R, Zhu H, An YQ, Beers EP, Liu Z (2012). Apple *miRNA*s and tasiRNAs with novel regulatory networks. Genome Biol..

[79] Adai A, Johnson C, Mlotshwa S, Archer-Evans S, Manocha V, Vance V, Sundaresan V (2005). Computational prediction of *miRNA*s in Arabidopsis thaliana. Genome Res..

[80] Zhang L, Chia JM, Kumari S, Stein JC, Liu Z, Narechania A, Maher C, Guill K, McMullen M, Ware D (2009). A genome-wide characterization of microRNA genes in maize. PLoS Genet..

[81] Joshi T, Yan Z, Libault DM, Jeong H, Park S, Green PJ, Sherrier DJ, Farmer A, May G, Meyers BC (2010). Prediction of novel *miRNA*s and associated target genes in *Glycine max*. BMC Bioinform..

[82] Szklarczyk D, Gable AL, Lyon D, Junge A, Wyder S, Huerta-Cepas J, Simonovic M, Doncheva NT, Morris JH, Bork P, Jensen LJ (2019). STRING v11: protein–protein association networks with increased coverage, supporting functional discovery in genome-wide experimental datasets. Nucl. Acids Res..

[83] Xu L, Dong Z, Fang L, Luo Y, Wei Z, Guo H, Zhang G, Gu YQ, Coleman-Derr D, Xia Q, Wang Y (2019). OrthoVenn2: a web server for whole-genome comparison and annotation of orthologous clusters across multiple species. Nucleic Acids Res..

[84] Babicki S, Arndt D, Marcu A, Liang Y, Grant JR, Maciejewski A, Wishart DS (2016). Heatmapper: web-enabled heat mapping for all. Nucl. Acids Res..

