# Supplementary material for: Genome wide in-silico miRNA and target network prediction from stress responsive Horsegram (Macrotyloma uniflorum) accessions
Source: Sci Rep. 2020 Oct 14;10:17203. doi: 10.1038/s41598-020-73140-x (PMC7560861; doi:10.1038/s41598-020-73140-x)
Supplement: Supplementary file 1 — Supplementary Table. [file 41598_2020_73140_MOESM1_ESM.docx]

**Genome wide *in-silico miRNA* and target network prediction from stress responsive Horsegram (*Macrotyloma uniflorum*) accessions**

Jeshima Khan Yasin ^1†^ Bharat Kumar Mishra^1,2†^, M. Arumugam Pillai^3^, Nidhi Verma^4^, Shabir H. Wani^5^, Elansary HO^6,7^, El-Ansary DO^8^, Pandey PS ^9^,

and Viswanathan Chinnusamy^10^

1. Division of Genomic Resources, ICAR-National Bureau Plant Genetic Resources, PUSA campus, New Delhi- 110012 INDIA Email: [Yasin.Jeshima@icar.gov.in](mailto:Yasin.Jeshima@icar.gov.in)
2. Present address: Department of Biology, University of Alabama at Birmingham, AL, USA AL 35294-1170 [bharat26@uab.edu](mailto:bharat26@uab.edu)
3. Department of Plant Breeding and Genetics, Agricultural College and Research Institute, Tamil Nadu Agricultural University, Killikulam, Vallanadu, Tamil Nadu- 628252, INDIA [mapillai1@hotmail.com](mailto:mapillai1@hotmail.com)
4. Principal Scientist (Education Planning & Home Science), Agricultural Education Division Krishi Anusandhan Bhawan I, Indian Council of Agricultural Research, PUSA Campus, New Delhi 110 012 INDIA

Mountain Research Centre For Field Crops, Khudwani Anantnag-192101, Sher-e-Kash*miR* University of Agricultural Sciences and Technology of Kashmir, J&K, INDIA [shabirhussainwani@gmail.com](mailto:shabirhussainwani@gmail.com)

1. Plant Production Department, College of Food and Agricultural Sciences, King Saud University, P.O. Box 2455, Riyadh 11451, Saudi Arabia; [helansary@ksu.edu.sa](mailto:helansary@ksu.edu.sa)
2. Floriculture, Ornamental Horticulture, and Garden Design Department, Faculty of Agriculture (El-Shatby), Alexandria University, Alexandria 21545, Egypt
3. Precision Agriculture Laboratory, Department of Pomology, Faculty of Agriculture (El-Shatby), Alexandria University, Alexandria, Egypt; [diaaagri@hotmail.com](mailto:diaaagri@hotmail.com)
4. Assistant Director General , Indian Council of Agricultural Research (ICAR), PUSA, New Delhi - 110 012, INDIA [adgephs@gmail.com](mailto:adgephs@gmail.com), [pspandey@icar.org.in](mailto:pspandey@icar.org.in)
5. Principal Scientist & Head, Division of Plant Physiology, Indian Agricultural Research Institute, New Delhi-110012, INDIA [viswanathan@iari.res.in](mailto:viswanathan@iari.res.in)

| S.No | Primer Name | Primer sequences |
| --- | --- | --- |
| 1 | 1F | GGTATGGGAGGTGTA |
| 2 | 2F | TTCCTTCCCAATCCC |
| 3 | 3F | AGAATTTGTGGGAAT |
| 4 | 4F | GGAATGGGCTGATTG |
| 5 | 5F | TCTTCCCAATTCCGC |
| 6 | 6F | TGCTCTCTATCTTCT |
| 7 | 7F | TCTTCCTCTTCCTCT |
| 8 | 8F | TGAGTTGAGTTGAGT |
| 9 | 9F | GACAGAAGAGAGAGA |
| 10 | 10F | AGACGATGTATGGAA |
| 11 | 11F | TCGCTTGGTGCAGGT |
| 12 | 12F | AGGAATGGGTGGAAT |
| 13 | 13F | AGGAATGGGTGGAAT |
| 14 | 14F | GTGCTATCCCTCCTG |
| 15 | 15F | GCGCTATCCCTCCTG |
| 16 | miRNA Univ R | CAGGAAACAGCTATGACCTTTTTTTTTTTTTTTTTTTTTTTTTTTT |
